# Surface Integrity and Mechanical Performance of Vertically Printed FDM-PLA After Laser Polishing: Coupled Effects of Scan Orientation and Hatch Distance

**DOI:** 10.3390/polym18202559

**Published:** 2026-10-17

**Authors:** Gabriel Stolárik, Radoslav Vandžura, Svetlana Radchenko, Petr Hlaváček, Matúš Geľatko

**Affiliations:** 1 Faculty of Manufacturing Technologies with a Seat in Prešov, Technical University of Kosice, Štúrova St. 31, 080 01 Prešov, Slovakia; radoslav.vandzura@tuke.sk (R.V.); svetlana.radchenko@tuke.sk (S.R.); matus.gelatko@tuke.sk (M.G.); 2 Institute of Geonics, The Czech Academy of Sciences, Studentska 1768/9, 708 00 Ostrava, Czech Republic; petr.hlavacek@ugn.cas.cz

**Keywords:** post-processing, laser polishing, scanning orientation, hatch distance, modification, roughness, strength, fracture

## Abstract

Vertically printed fused deposition modelling (*FDM*) components exhibit pronounced surface irregularities on their sidewalls at the interfaces between deposited layers, while limited interlayer bonding can impair their tensile performance. Although laser polishing has shown potential to improve the surface quality of vertically printed *FDM* components, the combined influence of laser scan orientation relative to the printed layers and hatch distance on the surface integrity and mechanical response of *PLA* remains insufficiently understood. To address this gap, a novel methodology for determining hatch distance (*H*) is introduced, with *H* defined as 25%, 50%, 75%, and 100% of the experimentally measured groove width (*GW*). To maximize structural and surface performance, three scanning strategies, *parallel* (0°), *perpendicular* (90°), and *oblique* (45°) to the layer interfaces, were evaluated using ANOVA, regression models, and SEM analysis. The results demonstrate a fundamental synergy between scan orientation and melt-flow dynamics. At *H* = 25%, *perpendicular* scanning produced the largest *Sa* reduction (59%), whereas *parallel* scanning yielded the lowest *Sdr* (3.14%, compared with 20.74% for the as-printed raw surface). At the same *H*, the *Sdr* was 4.45% for *perpendicular* and 8.45% for *oblique* scanning. *SEM* analysis indicates that while all strategies eliminated the inherent layered texture, the *oblique* strategy (45°) additionally led to more thorough valley filling through melt flow from two adjacent peaks. Specific surface porosity was identified at modified interfaces, providing indications of gas entrapment and bubble expansion within the heat-affected zone (*HAZ*). Statistical height distribution (positive *Ssk* and *Sku* < 3) demonstrates that *HAZ*-driven surface rounding occurs across all orientations. The greatest improvement in tensile performance was observed with *oblique* scanning (45°) at *H* = 100%, where peak force increased by 11.2% relative to that of the raw material. Fractography indicates that this improvement originates from a preserved ductile skin-layer that delays delamination, shifting the failure mode toward localized internal fibre breakage. This work establishes scanning orientation as the primary determinant of topography, while hatch distance dictates homogenization intensity, offering a systematic framework for optimising the laser polishing of polymers.

## 1. Introduction

Additive manufacturing (*AM*) is a dynamically developing field that offers significant advantages, especially in the context of rapid prototyping and production of geometrically complex structures [1,2]; thus, it is included in the advanced Industry 4.0 concept [3]. Multilayer construction is produced using several methods, which can be divided according to ISO/ASTM 52900:2021 [4] into binder jetting (*BJT*), directed energy deposition (*DED*), material extrusion (*MEX*), material jetting (*MJT*), powder bed fusion (*PBF*), sheet lamination (*SHL*) and vat photopolymerization (*VAT*), which include traditionally known methods such as fused deposition modelling (*FDM*), stereolithography (*SLA*), selective laser sintering/melting (*SLS/SLM*) [5] and various others [6]. The most common *AM* methods include the *FDM* technique, in which the extruded molten material is deposited layer-by-layer along a precisely defined path, by which the required shape of the part is created [7]. *FDM*, also referred to as fused filament fabrication (*FFF*), can process a range of thermoplastics, including polylactic acid (*PLA*), acrylonitrile butadiene styrene (*ABS*), glycol-modified polyethylene terephthalate (*PETG*), polyamide (*PA*), polycarbonate (*PC*), and thermoplastic polyurethane (*TPU*) [8]. Among these materials, *PLA* is widely used owing to its favourable strength [9] and stiffness, as well as its thermal processing characteristics [10]. Its bio-based origin and potential for recycling also make it relevant to sustainable material development [11]. Recent advances have expanded *FDM* feedstocks to composite filaments containing natural fibres [12] and wood-based fillers [13], ceramic and metallic particles, and carbon-based additives, alongside recycled polymers and other bio-based or functional constituents [14,15]. If the parts produced using *FDM* serve as an exact copy of the part in the prototyping process or an active part of the assembly, then geometric accuracy [16], surface quality [17] and strength are important aspects of their application. The geometric accuracy depends on the printing parameters, and the functionality depends on the surface characteristics or strength of the created component. In *FDM*, the surface roughness is influenced by the parameters of the last, or outer, deposited layer, which defines the outer part of the component [18]. However, the modelling of parts is associated with their construction in the *z*-axis, where the surface quality is significantly influenced by the outer layers of individual stairs at shaped contours [19]. It has been proven that changing the orientation of the build [20,21] has a direct impact on the surface quality of the printed part in vertical orientation [22]. Considering the mechanical properties (strength), defects on the interfaces of deposited layers within the printing process [23] reduce the structural integrity and cause cracks, which consequently lead to fatigue failure [24]. Therefore, several surface treatment methods have been proposed with the objective of strengthening the surface by reducing the roughness and improving its mechanical properties [25]. It has been shown that laser treatment can strengthen and controllably harden the surface and reduce the roughness without tool wear [26], which makes this method attractive for its real application [27]. Table 1 shows an overview of studies dealing with laser post-processing of *FDM* materials and their main achieved results.

Research in the field of laser post-processing of *FDM* prints confirms the critical influence of the induced energy density (*ED*) on the resulting surface topography. A study [28] reports that the roughness *Ra* is reduced by 67–95% at an *ED* in the range of 5–11 J∙cm^−2^. Similar efficiency for *PLA* materials was confirmed by the authors in [29], where a reduction of the *Sa* parameter by 67.83% was achieved by correctly setting the laser power (*P* = 3 W) and hatch distance (*H* = 0.05 mm), in combination with scan speed (*vs* = 150 mm∙s^−1^). In addition to the power parameters, the beam geometry also plays a key role. Research with fibre lasers (*FL*) [30] has shown that a larger focal diameter (200 µm) achieves a 2–8% higher smoothing rate than that using a smaller diameter (175 µm) at identical powers (3–4 W). This phenomenon is related to the energy distribution and the ability of the melt to fill the gaps between the layers more efficiently, which leads to an increase in the elastic modulus by 16.1% and an increase in tensile strength up to 52.98 MPa compared to the 39.42 MPa of the raw samples [32]. Laser polishing also eliminates the risk of delamination of the layers due to the remelting of surface and subsurface structures [31]. The process is further significantly limited by the scanning speed (*vs*). In the study [33], a nonlinear dependence was observed, where a decrease in the speed from 200 to 50 mm·s^−1^ initially reduces the roughness, but after exceeding the threshold value, excessive heat input leads to melt spattering and material ablation [34]. The efficiency of the interaction is also affected by the focal length [35], which, in combination with the hatch distance *H*, determines the areal density of the induced energy. Finally, the optical and structural properties of the print also contribute to the process, where the absorption of laser energy is directly dependent on the material pigmentation [36] and the infill density (*ID*). Furthermore, research [37] shows that samples with lower infill density (*ID*) can exhibit lower roughness after laser polishing (*Ra* = 12.07 μm to *Ra* = 1.96 μm at *ID* = 90% and *ID* = 40%, respectively) due to the better ability of the melt to flow into the internal cavities, which was also observed in [38], and which leads to the elimination of the surface waviness characteristic of fully dense structures. This also affects the surface wettability, where the change in surface quality affects the resulting contact angle [39]. Simultaneously, wet polishing techniques can significantly reduce the risk of thermal degradation of the material, making the process more stable [40]. Hence, optimisation of polishing processes becomes an essential area of research from the perspective of accelerating the polishing process and the ability to predict material response [41].

Previous studies have demonstrated that laser post-processing can modify the surface integrity of *FDM* parts through the optimization of processing parameters. For parts built along the *z*-axis, however, the sidewall topography is defined by the exposed contours of individual deposited layers, while mechanical strength depends critically on the quality of interlayer bonding [20,21,23]. Most previous laser-polishing studies have focused on horizontally printed parts and have shown that hatch distance (*H*) and the resulting track overlap affect surface quality. In vertically printed parts, insufficient overlap can divide the layered surface into distinct microreliefs [42]. These features increase surface roughness and may act as stress concentrators that impair mechanical performance [42]. Although a previous study identified this problem in vertically printed *PLA*, it used a fixed track spacing and scan direction and therefore, did not establish how these parameters should be jointly selected [42]. Whereas [42] examined laser power and scanning speed at a fixed hatch distance and a single scan orientation, the present study evaluates three scan orientations and four programmed hatch distances under constant nominal areal energy density. Moreover, studies investigating *H* for horizontally or partially tilted printed surfaces [29,35,39,40] do not provide a clear basis for selecting hatch distance in relation to the exposed layer structure of vertically printed *PLA*. Thus, how hatch distance and laser scan orientation jointly affect both surface integrity and mechanical response in this configuration remains insufficiently understood. To address this gap, the present study measures the actual width of a single laser track and defines *H* as 25%, 50%, 75%, and 100% of that width. These hatch distances are evaluated with scan paths *parallel* (0°), *perpendicular* (90°), and *oblique* (45°) to the printed layers. The resulting comparison provides a systematic basis for assessing the effects of track spacing and scan orientation on the surface topography and mechanical performance of vertically printed *PLA*.

## 2. Materials and Methods

### 2.1. Experimental Material

For experimental purposes, a commercial grey *PLA* filament (ELEGOO PLA Matte; 1.75 mm diameter; 1 kg spool; label identifiers 1-2506-0605 and 50.203.0256, Shenzhen Elegoo Technology Co., Shenzhen, China) was selected. The manufacturer specifies a printing-temperature range of 190–230 °C. The detailed composition of this commercial matte filament was not independently determined; therefore, the presence or absence of pigments and other additives cannot be established. Prior to printing, the filament was dried in a CREALITY (Shenzhen Creality 3D Technology Co., Shenzhen, China) filament dryer at 50 °C for 6 h. The reported value of 8% refers to the relative humidity inside the dryer chamber, not to the moisture content of the filament. The same grey filament spool was used for all specimens, avoiding variation associated with differences in filament colour and formulation [36]. The geometry of the experimental samples was designed in accordance with the ISO 527-2:2012 standard (type 1B) [43]. The samples were placed in a vertical orientation along the *z*-axis (150 mm height), with a cross-section in the XY plane of 10 × 4 mm. This orientation was intentionally selected to generate a characteristic surface texture with a distinctive staircase effect.

The samples were printed using a Bambu Lab (Shenzhen Tuozhu Technology Co., Shenzhen, China) X1 Carbon (X1C) printer with an enclosed chamber. Printing parameters were set in Bambu Studio (version 2.3.0.15) using a modified “0.20 mm Standard @ BBL X1C” process profile and a “Generic PLA” filament profile. A standard 0.4 mm nozzle was used, and both the first-layer height and subsequent layer height were set to 0.20 mm. The nozzle temperature was 205 °C, and the build-plate temperature was 60 °C. The specimens were printed vertically along the *z*-axis. Five wall perimeters and an infill density of 0% were specified. The seam position was set to “aligned”, resulting in a consistent seam location along the specimen height. A brim was used to improve build-plate adhesion and printing stability. The specimens were produced in successive batches of ten using the same printer and printing settings. Detailed printing parameters, specimen geometry and specimen quality are presented in Table 2.

### 2.2. Experimental Method

**Figure 1:**
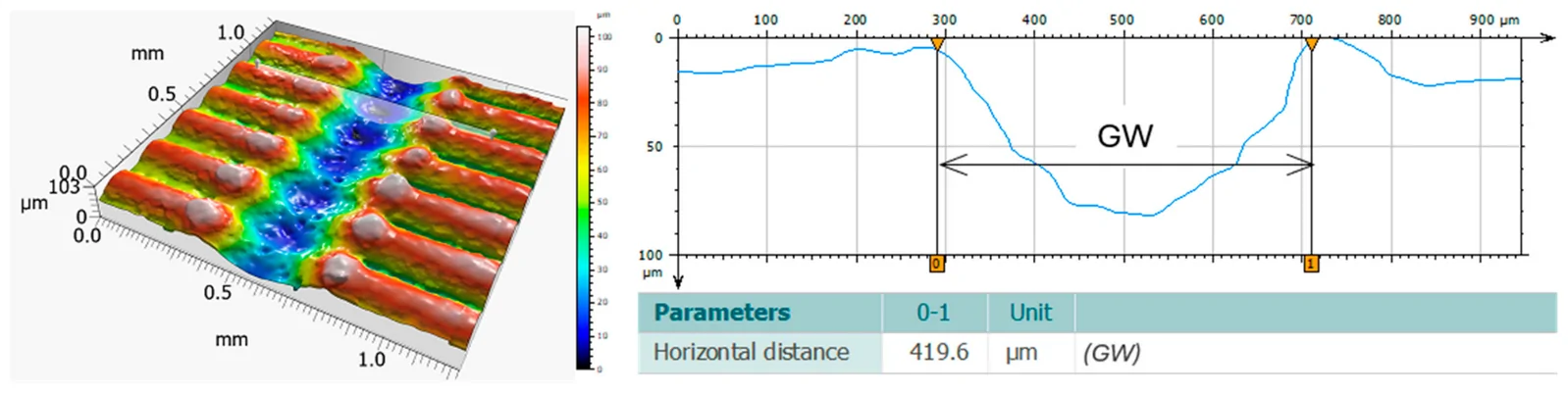
Measured groove width in pre-experimental setting.

**Figure 2:**
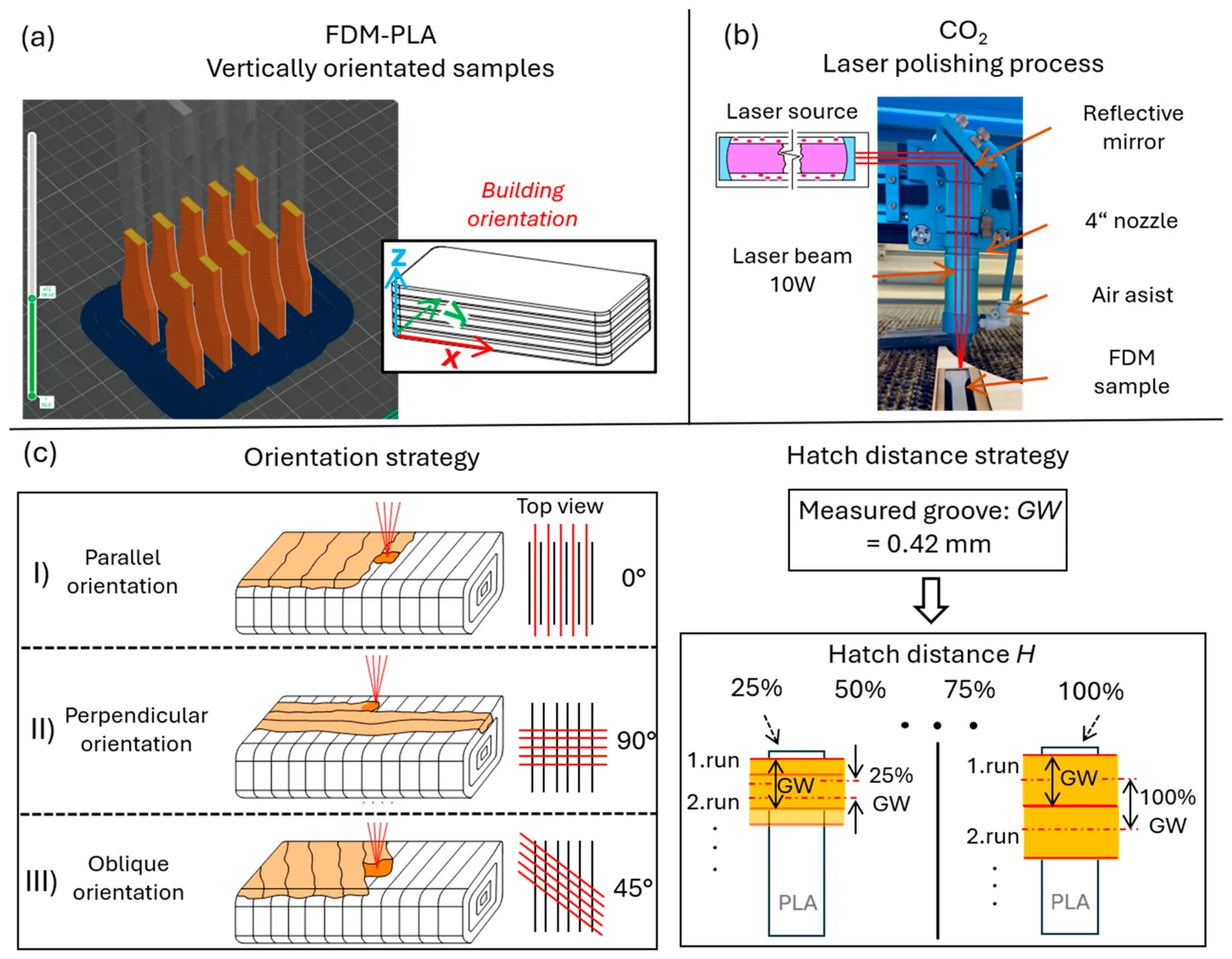
Experimental setup: (**a**) printing orientation, (**b**) laser technology, (**c**) orientation strategy and hatch distance determination.

The laser polishing process was carried out on a Thunder Laser Nova 51 device (Thunder Laser Industrial Co., Ltd, Dongguan, China) equipped with a CO_2_ source, with a maximum power of 100 W and active liquid cooling of the tube. A laser beam with a primary diameter of ~6.35 mm and a wavelength of ~10.6 µm was guided by a system of reflective mirrors into a process head equipped with a 4″ focusing lens (Figure 2b). The configuration provided a theoretical spot focus of ~194 µm at a nominal focal length of 101.6 mm. Before the experiment itself, the working distance (*WD*) between the nozzle tip and the material surface was optimised in the range of 10–24 mm in order to define the real width of the trace and minimise the thermal degradation. Based on preliminary tests, the optimal distance *WD* = 20 mm (defocusing) was determined. With parameters *P* = 10 W and *vs* = 150 mm·s^−1^ selected based on [42], this configuration showed effective remelting of roughness peaks without signs of degradation of the material’s lower layers. The reference groove width (*GW*) was set to 419.6 µm for these conditions (Figure 1).

In each case, a high-pressure air assist (with a maximum pressure of 6–7 psi, as specified by the manufacturer) was used during the process, which minimises the risk of excess absorption of laser energy by vapours and additional overheating of the material; therefore, a protected interaction of the beam with the material and elimination of its degradation was ensured. Consequently, faster solidification prevents deformation of the sample geometry, the preservation of which is essential for subsequent evaluation of mechanical properties (tensile tests).

The main focus of the research was analysing the influence of laser polishing orientation and transverse offset (Hatch distance *H*) on the structural integrity of vertically printed samples (Figure 2c). The experiment was divided according to the direction of the laser paths with respect to the orientation of the print layers:

I. *Parallel orientation* (0°): the polishing direction is parallel with the orientation of the layers.
II. *Perpendicular orientation* (90°): the polishing direction is perpendicular to the orientation of the layers.
III. *Oblique orientation* (45°): polishing direction is at a defined angle to the orientation of the layers.

Within each orientation, the parameter *H* was varied, defined as the percentage of the reference groove width of 25%, 50%, 75% and 100% (Figure 2c) of the measured *GW* = 419.6 µm (Figure 1). In all cases, the samples were processed across their entire surface area, on both the top and bottom sides. Laser processing was carried out in a single pass along a programmed path generated using ThunderLaser v8 software (2021), which is fully compatible with the ThunderLaser system.

The *H* range was designed to cover the region of significant track overlap (25–75%), where the highest surface homogeneity is assumed, up to the region of isolated tracks (100%). In order to isolate the influence of the scanning geometry, the scanning speed (*vs*) was linearly adjusted at the transverse change of displacement. This procedure maintained a constant nominal areal energy density (*ED*), calculated using Equation (1):

(1) $$\mathrm{ED}=\frac{P}{\mathrm{vs}\cdot H}\;[J\cdot {\mathrm{mm}}^{-2}]$$

where *P* is the nominal laser power, *vs* is the scanning speed, and *H* is the hatch distance.

In this configuration, however, the energy delivered per unit length of each laser path varied. Changing the scanning speed between 150 and 600 mm·s^−1^ changed the beam interaction time and the line energy (*EL*), calculated using Equation (2):

(2) $$\mathrm{EL}=\frac{P}{\mathrm{vs}}\;[J\cdot {\mathrm{mm}}^{-1}]$$

Ultimately, this leads to an experimental setup win which, while maintaining the surface energy density (*ED*), the parameter *H* and the transition orientation were isolated—albeit with a concurrent change in the local linear energy density, which could have influenced the surface melting process of the *PLA*. The tests were repeated five times for each change in the experimental parameter *H* or the orientation of the transition in order to obtain statistical data. Detailed values of the technological parameters are shown in Table 3.

**Figure 3:**
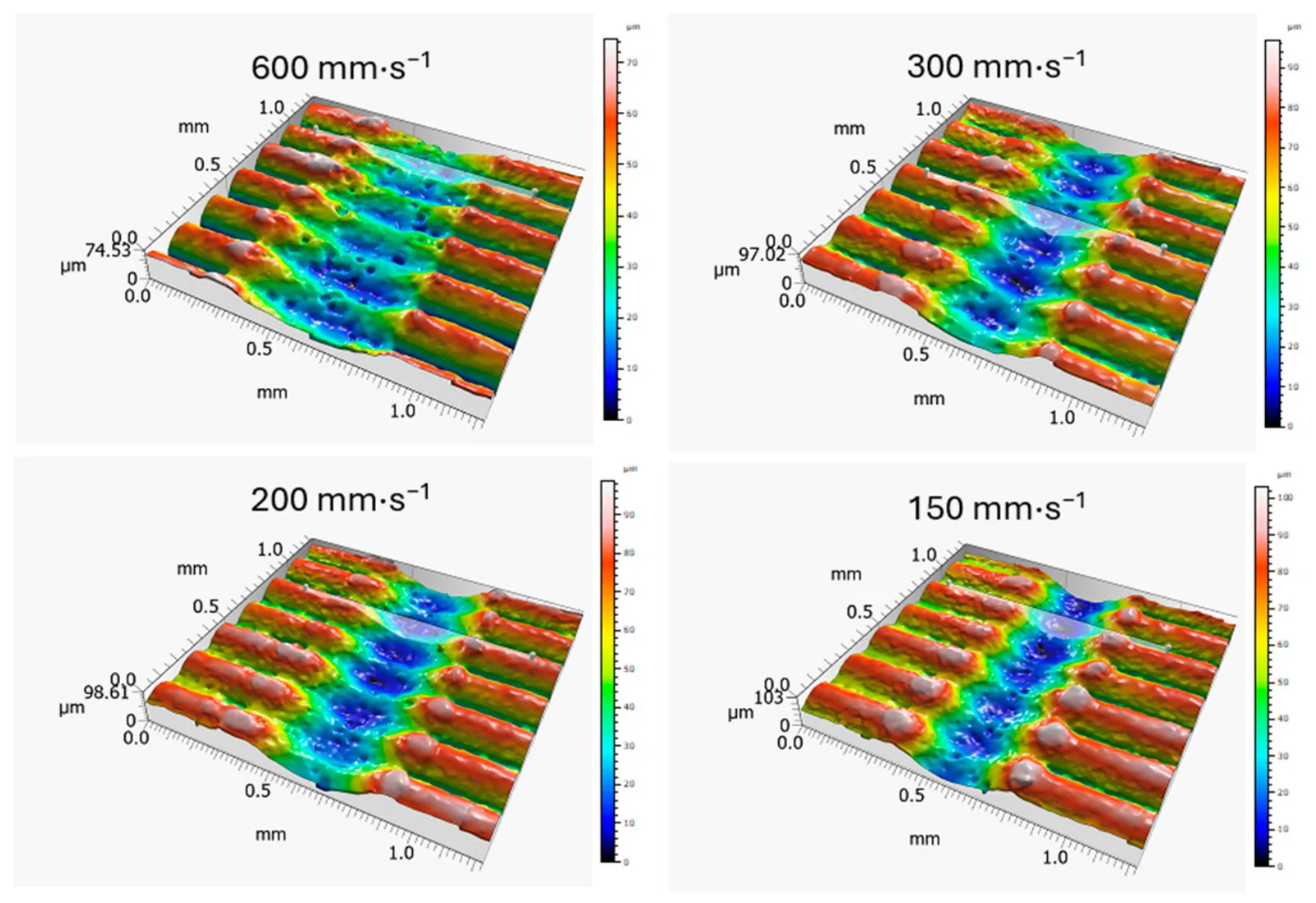
Optical analysis of the groove after laser processing at 10 W and speeds of 150, 200, 300, and 600 mm·s^−1^.

The reference groove width (*GW* = 419.6 µm), used to define the programmed hatch distance *H*, was determined from a single laser track produced at *vs* = 150 mm·s^−1^. This speed was selected because the topographic map showed a more continuous reduction in the original interlayer relief within the laser-modified region than did the maps obtained at 200, 300, and 600 mm·s^−1^ (see in Figure 3). At the higher speeds, shorter beam interaction times and lower line density energy (*EL*) were accompanied by more visible remnants of the printed-layer texture. This was defined as insufficient in terms of suppressing the original layered structure. The measured apparent track widths were 513, 551, and 567 µm at 200, 300, and 600 mm·s^−1^, respectively.

Thus, the *H* values of 25%, 50%, 75%, and 100% represent fractions of *GW* rather than speed-independent track overlap ratios. Using the track width measured at each speed, the estimated geometric overlaps were 81.5% for *H* = 25% (600 mm·s^−1^), 61.9% for *H* = 50% (300 mm·s^−1^), 38.6% for *H* = 75% (200 mm·s^−1^), and approximately 0% for *H* = 100% (150 mm·s^−1^). For clarity, the manuscript presents the overlap based on the measured GW at 150 mm·s^−1^, calculated from the theoretical values for this setting.

### 2.3. Measurement Method

The as-printed sample, together with the laser post-processing samples, were analysed using optical 3D imaging, based on the scans obtained using a KEYENCE VHX-7000 digital microscope (KEYENCE Corporation, Osaka, Japan). The samples were scanned at the central part of the shaft, with a scan area of 15 × 4 mm. Scanning was performed at 300× magnification; each sample was scanned once, and with the experiment repeated five times, this enabled five replicate roughness measurements for the given process parameters. The scans were processed in Mountains SPIP 11 expert version software after filtering the *S* and *F* filter, in accordance with the ISO 25178-2 standard [44]. During sample processing, the scans were aligned using the Total Least Squares Plane (TLSPL) method, and the “Fill NM points” filter was applied to address missing data points. For optical analysis, the scans were cropped into uniform 4 × 4 mm squares to enhance the visibility and resolution of surface irregularities. The topography of the surface height roughness parameters *Sa*, *Sz*, *Ssk*, *Sku*, hybrid parameter *Sdr*, and functional parameters *Spk* and *Svk* were analysed. The roughness parameters were statistically and graphically processed as the average value ± standard deviation. The measurement results were further statistically processed in Minitab software (version 22) in terms of the normality test (probability plot), regression analysis and ANOVA. The mechanical properties were examined using the Testometric X350 universal testing machine (Testometric Co., Ltd., Rochdale, UK), in accordance with the ISO 527-2 standard-2 [43]. Tensile tests were performed at a standard laboratory temperature of 21 °C and a humidity of 55 ± 5%. The test loading rate was set to 20 mm∙min^−1^, in accordance with the ISO 527-2 standard-2 for thermoplastics. The mechanical properties were statistically processed as the mean ± standard deviation. Elongation was derived from the crosshead displacement of the testing machine; no extensometer was used. Morphological changes were recorded using a Phenom XP G3 scanning electron microscope (Phenom-World BV, Eindhoven, The Netherlands), with an acceleration voltage of 5 kV under a vacuum of 10 Pa. Before *SEM* analysis, the samples were coated with a 3 nm gold layer using an automated Luxor Au device (Aptco Technologies GmbH, Nazareth-De Pinte, Belgium) in an argon environment.

## 3. Results and Discussion

In this section, the achieved sample quality results are presented and discussed in correlation with the individual stages of the experimental process. Morphological changes of the surface were characterised using 3D digital reconstruction of the scans, supplemented by subsequent optical analysis of representative samples from each experimental set. For detailed identification of the physical mechanisms of the process, the morphology was also analysed using scanning electron microscopy (*SEM*), which provides detailed information about the interaction of the laser beam with the material. The quality of the modified surfaces is further quantified using surface roughness parameters, which served as input data for ANOVA and regression analysis in order to identify the most significant influences of the technological factors. In the final Section 3.4, the mechanical properties of the samples are evaluated based on the monitored post-processing parameters, which allows for a more comprehensive resulting integrity assessment of the samples.

### 3.1. Optical Analysis

**Figure 4:**
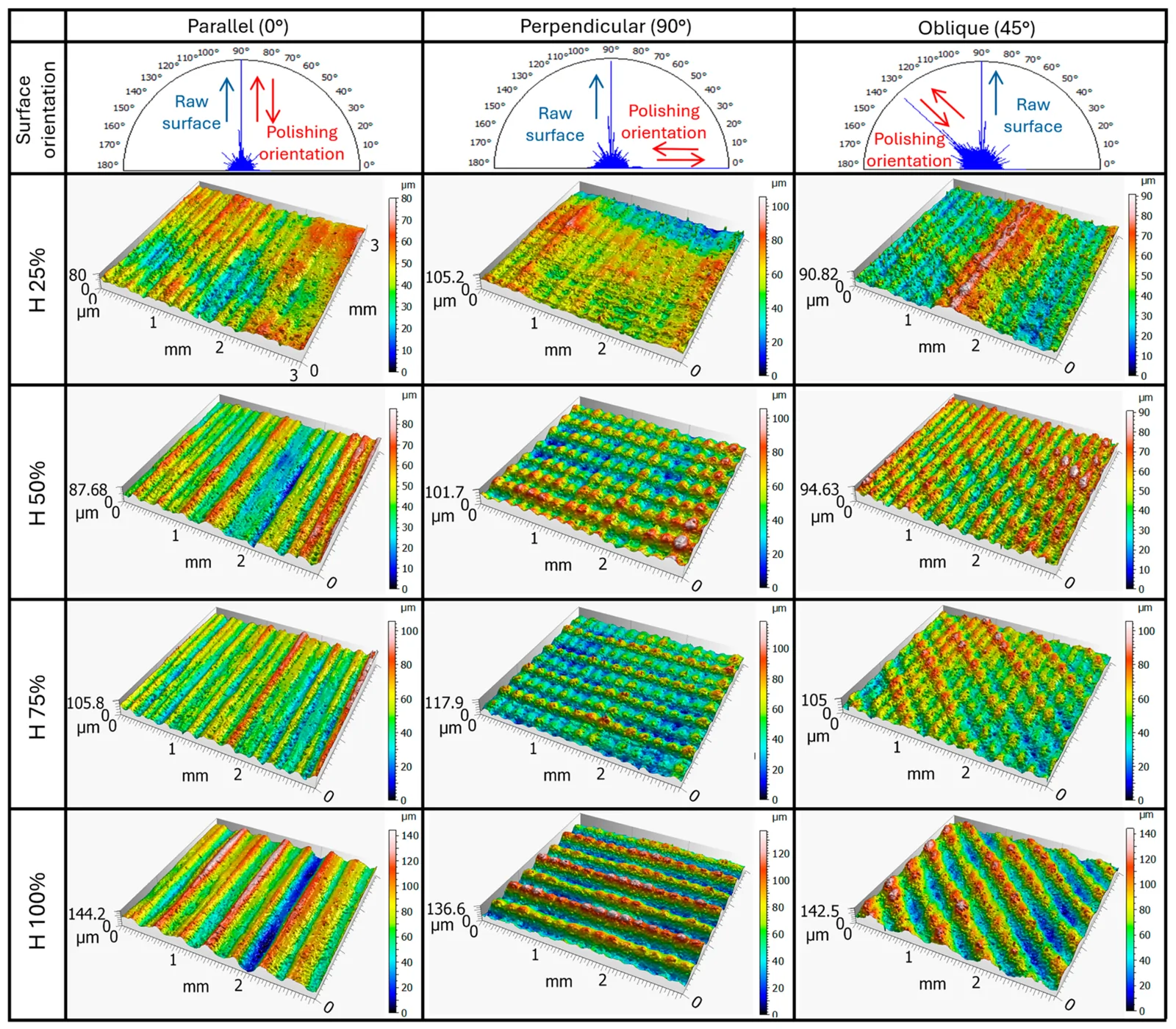
Surface orientation analysis and optical analysis of *FDM* samples after laser post-processing.

The optical analysis (Figure 4) confirmed that each laser post-processing mode induces significant changes in the surface morphological characteristics. The directional texture distribution, verified by angular analysis, demonstrated a direct dependence of the topography on the selected scanning orientation. Because hatch distance and scanning speed were varied together to maintain a constant nominal areal energy density, differences in surface morphology may reflect both scan hatch distance and the accompanying change in line energy.

*Parallel orientation* (0°) shows a dominant directionality in one axis, which is given by the identical orientation of the laser paths with the original orientation of the print layers. The highest level of surface homogenization was recorded at *H* = 25% (*EL* = 0.167 J·cm^−1^). Considering a layer thickness of 0.2 mm and the beam shift at a value of 0.105 mm (approximately half the peak spacing), each peak of the layer was exposed to laser radiation at least twice. The slight waviness of the surface at this setting is probably a consequence of the redistribution of the melt, where the material from the peaks flows into the interlayer valleys due to gravity [33,37]; moreover, when the beam passes directly through the centre of the valley, it can be locally deepened. Increasing the shift to *H* = 50% (0.21 mm, *EL* = 0.33 J·cm^−1^) led to the appearance of alternating linear maxima and minima. This phenomenon is caused by the fact that the hatch distance is nearly correlated with the layer thickness, which leads to “skipping” of the peak lines or their melting into adjacent valleys during direct hit by the beam axis. At *H* = 75% (*EL* = 0.500 J·cm^−1^) and *H* = 100% (*EL* = 0.667 J·cm^−1^), the process is unpredictable due to geometric mismatch (interference of the 0.315 mm and 0.42 mm distances relative to the 0.2 mm layer). At *H* = 100%, a selective deepening of the original valleys is present if the beam enters the interlayer space, while the peaks remain negligibly thermally affected.

*Perpendicular orientation* (90°) generates a two-peaked texture distribution (printing direction vs. polishing direction). While at *H* = 25%, the surface appears visually unified, with increasing offset (*H* = 50–100%), it is segmented into relief structures [33]. This condition is caused by the non-uniform energy distribution in the beam cross-section (see Figure 1). Considering the rounded profile of a groove [45], with a decreasing temperature gradient away from the beam axis, even theoretical overlaps (*H* = 50% and 75%) do not ensure perfect smoothing. The result is the appearance of “gully” transitions that follow the laser scanning path.

*Oblique orientation* (45°) appears to be the most effective in terms of resulting homogeneity. At *H* = 100%, the two-peak topography is defined by the intersection of the original layers and the oblique laser paths; however, with decreasing displacement (*H* = 75–25%), this contrast is reduced due to a more uniform distribution of the thermal field. Importantly, the oblique orientation eliminates undesirable phenomena, such as the deepening of interlayer valleys (observed for 0°) and the formation of relief segmentation (observed for 90°), observed in previous strategies. Considering the view of optical morphology, it can be stated that the oblique orientation of 45° represents the optimal strategy for achieving a homogeneous surface on vertically oriented *FDM* samples.

### 3.2. SEM Analysis

**Figure 5:**
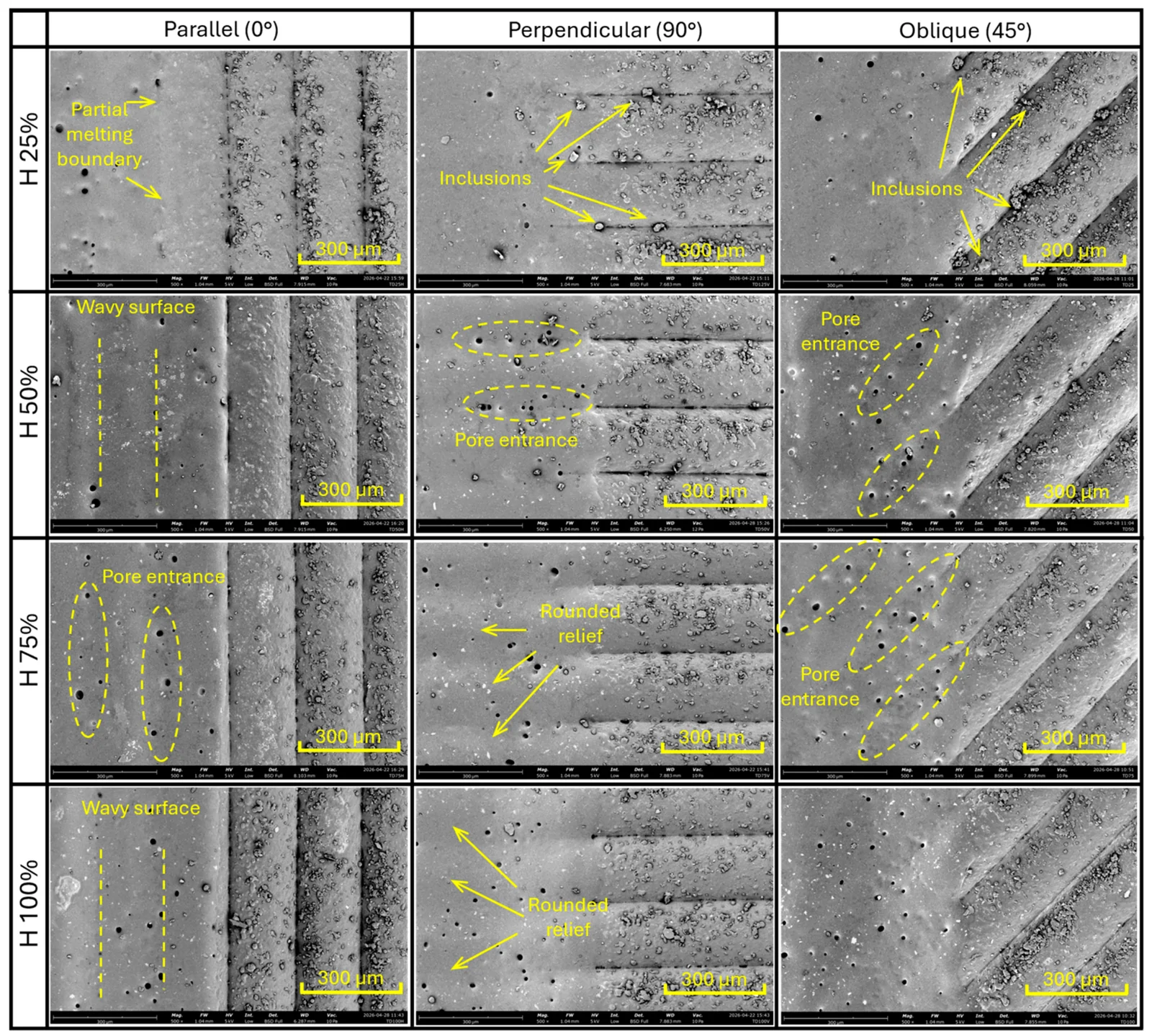
*SEM* analysis of *FDM* samples after laser post-processing.

Scanning electron microscopy (*SEM* Figure 5) analysis confirmed a fundamental transformation of the surface morphology for all laser polishing configurations. Regardless of the selected orientation or *H* parameter, continuous remelting and redistribution of the material (melt flow) were observed, leading to the elimination of the original layered texture of the *FDM* print, which was also observed in [30,36]. Although the polished surfaces exhibited broadly similar microgeometric features, the *SEM* images do not establish identical melt-pool conditions across the experimental sets. The scanning speed (*vs*) was adjusted with *H* to maintain a constant nominal areal energy density (*ED*); however, the resulting variation in line energy (*EL*) may also have influenced local remelting and melt redistribution.

In the case of *parallel orientation* (0°) at *H* = 25%, a smooth transition between the melting zone and the original surface is identifiable. A slight surface melting of the boundary peak is visible, which confirms the gradual and homogeneous thermal exposure of the material due to the fine-stepping (0.105 mm). During the subsequent passes, it can be assumed that these boundary lines would be completely covered and filled with melt by subsequent passes of the beam. In configurations of *H* = 50–100%, the boundaries of individual peaks are no longer identifiable, but the surface acquires a characteristic macroscopic waviness. This phenomenon correlates with the increasing hatch distance (0.21–0.42 mm), which initiates either a local deepening of the interlayer valleys or an insufficient volumetric redistribution of the melted peaks (described in the optical analysis).

*Perpendicular orientation* (90°) is characterised by the absence of the wavy surface characteristic of the *parallel* direction, which is a direct consequence of the *perpendicular* movement of the beam. However, the dominant feature is the fragmentation of the surface into rounded relief structures [33]. This morphological characteristic is attributed to the aforementioned uneven distribution of beam intensity in its cross-section, which previously led to a “gully” shape of a valley in a single pass [45]. Moreover, the rounding of the reliefs can be supported by partial melting of the surroundings during multiple passes due to the influence of the heat-affected zone *HAZ* [46], which can reach the distance of the previous pass and consequently affect the edges of the relief.

*Oblique orientation* (45°) does not show any signs of relief structures. It can be said that despite the uneven temperature distribution in the beam cross-section, the most homogeneous surface smoothing occurs in this orientation. This can be explained by the mechanism of multidirectional material flow, where the interlayer valleys are filled not only with the material of the currently melted surface, but also with the material from the subsequently melted adjacent peaks oriented at an angle of 45°.

Despite the overall smoothing, local surface openings resembling pores were observed on the modified surfaces (also observed in [31,33]), with several of them near the original interlayer interfaces (Figure 5). Their origin cannot be established from surface *SEM* images alone. Two possible, non-exclusive mechanisms are considered below: the reopening of pre-existing interlayer voids during remelting and the release of gaseous products during local heating.

- The laser polishing process generates a molten pool that gradually fills the macroscopic irregularities (valleys) of the *FDM* structure. However, the original interlayer joints contain air gaps due to the nature of the additive technology (see Table 2) [38]. Although the *PLA* melt covers these gaps due to gravity, the high viscosity of the polymer can prevent complete flow into the finest cavities [33]. While these gaps are covered, an internal air pore is created in the material. When the laser paths are subsequently overlapped, re-melting occurs, which can lead to the expansion of the air cavity and its release (deaeration) through the viscous layer. If the melt does not have time to fill the created channel again due to rapid solidification, a permanent defect in the form of a micropore is formed on the surface.
- An alternative explanation is local overheating of the material in the subsurface layers. Although the process is accompanied by an intense flow of air assist, the cooling effect is dominant mainly on the surface. With increasing depth and the presence of a continuous insulating layer of melt (at a high degree of overlap), the efficiency of heat removal decreases. When critical values of the temperature field are reached, gaseous products of thermal decomposition of *PLA* can be formed in the subsurface regions [33,47]. A tendency of these gases to escape through the viscous melt, in combination with the rapid cooling of the surface after the beam passes, leads to the “solidification” of the escape paths and the formation of pores in the final morphology. This type of pore could be a consequence of its formation outside the valleys of the interlayer connection.

These proposed mechanisms remain hypotheses; additional cross-sectional and thermal or chemical characterization would be needed to assess their contributions to the observed pore-like openings.

### 3.3. Roughness Analysis

**Figure 6:**
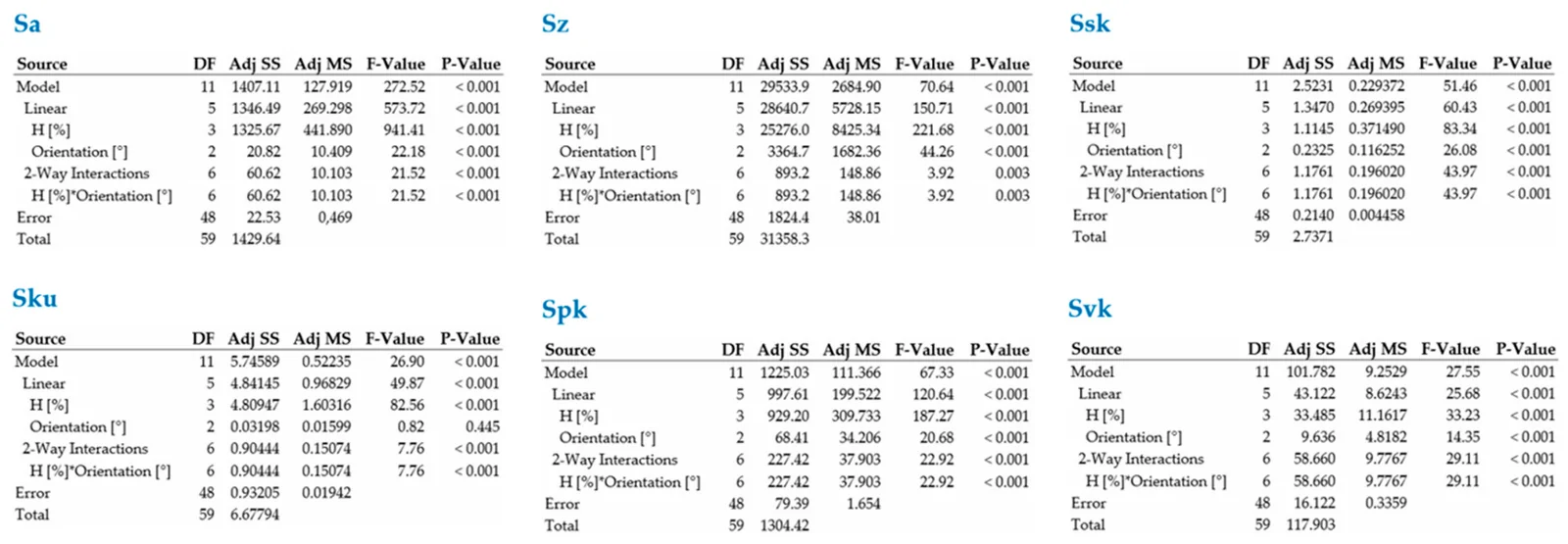
Analysis of Variance (ANOVA) for the *Sa, Sz*, *Ssk*, *Sku*, *Spk* and *Svk* parameters.

Selected roughness parameters were statistically analysed, whereas the experiment was conducted using a Design of Experiment (DoE) method. Within the multi-level factorial design, two input factors were represented by technological parameter *H* [%] and orientation [°], with 4 and 3 levels, respectively. As related responses, roughness parameters *Sa*, *Sz*, *Ssk*, *Sku*, *Spk* and *Svk* were monitored. The effect of the factors on responses was evaluated after five replicates; hence, 60 total runs were performed. The statistical significance was assessed using the Analysis of Variance (ANOVA) method, and the *p*-value was considered with respect to the level of significance α = 0.05. According to Figure 6, the effect of individual factors and their two-way interaction was significant for all evaluated responses, with the exception of the *Sku* parameter. The effect of orientation is higher than the level of significance α = 0.05. However, within its interaction with *H*, the *p*-value is lower than α. This can be ascribed to the characteristics of *Sku* (areal kurtosis), which is more significantly influenced by *H*, impacting the extent of local remelting of the layer peaks rather than the orientation.

**Figure 7:**
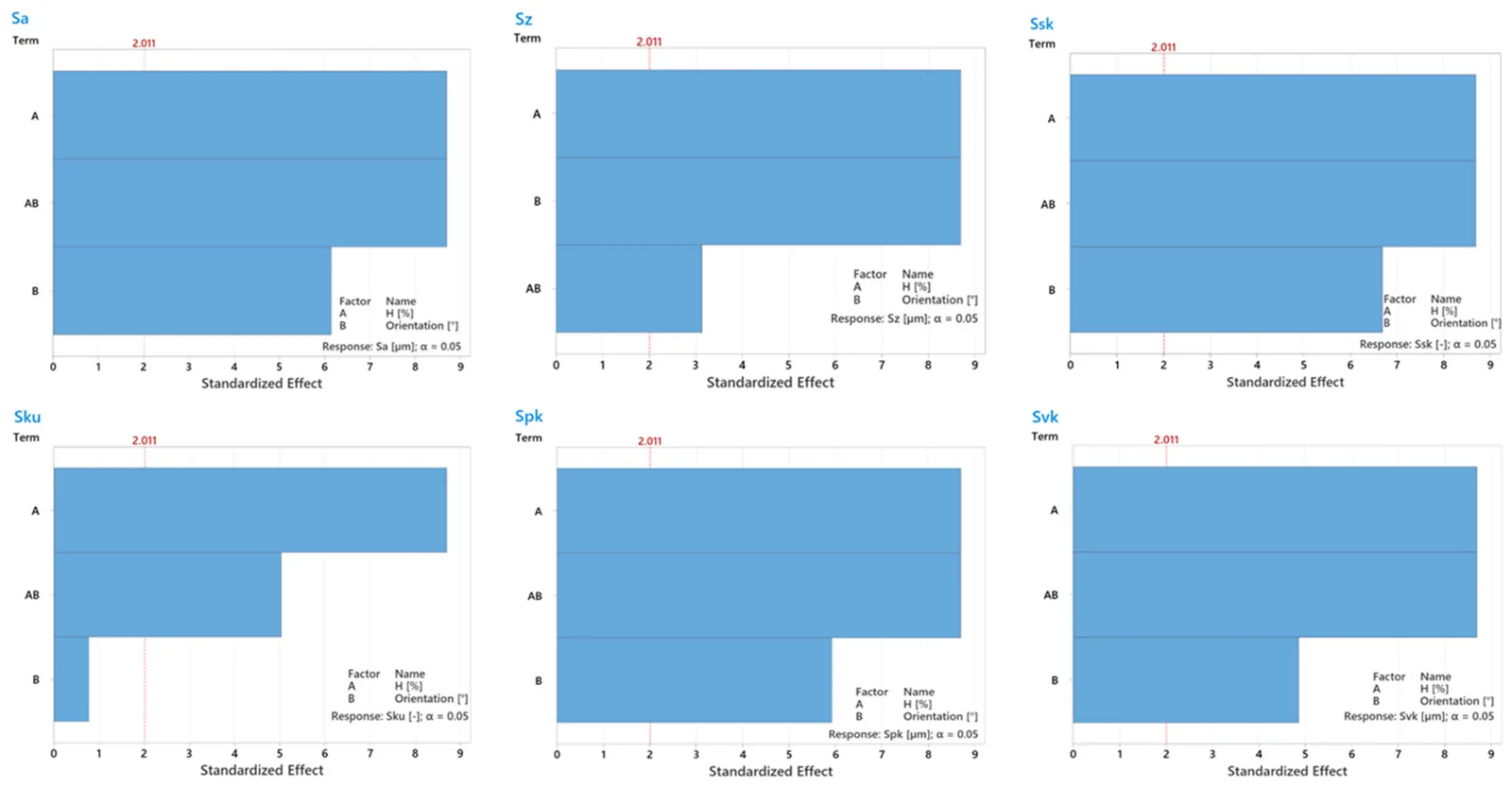
Pareto charts of standardized effects on the *Sa, Sz*, *Ssk*, *Sku*, *Spk* and *Svk* parameters.

Pareto charts of the standardized effect (Figure 7) were created for the interpretation of the effect of individual factors and their interaction on the responses (surface parameters). For all parameters, the A factor represented by *H* [%] proved to be the most influential. The AB interaction of *H* and orientation reached the same value (8.69) as the A factor in the case of the *Sa*, *Ssk, Spk* and *Svk* parameters. This can be related to the fact that beside the *H*, the orientation also significantly contributes to the arithmetical mean height, skewness, peak height and valley depth through the mechanism of melting the peaks and valleys. Factor B proved to be the least influential factor in the majority of surface parameters, particularly in the case of *Sku*, where it did not even reach the limit of the critical value of the standardized effect (2.011), as the consequence of a previously described scenario. A different order of factors was observed within the *Sz* parameter, where the orientation achieved the same effect as the *H*, although their interaction was less significant, which suggests that maximum height of surface area is not as dependent on the variances of the AB interaction.

**Figure 8:**
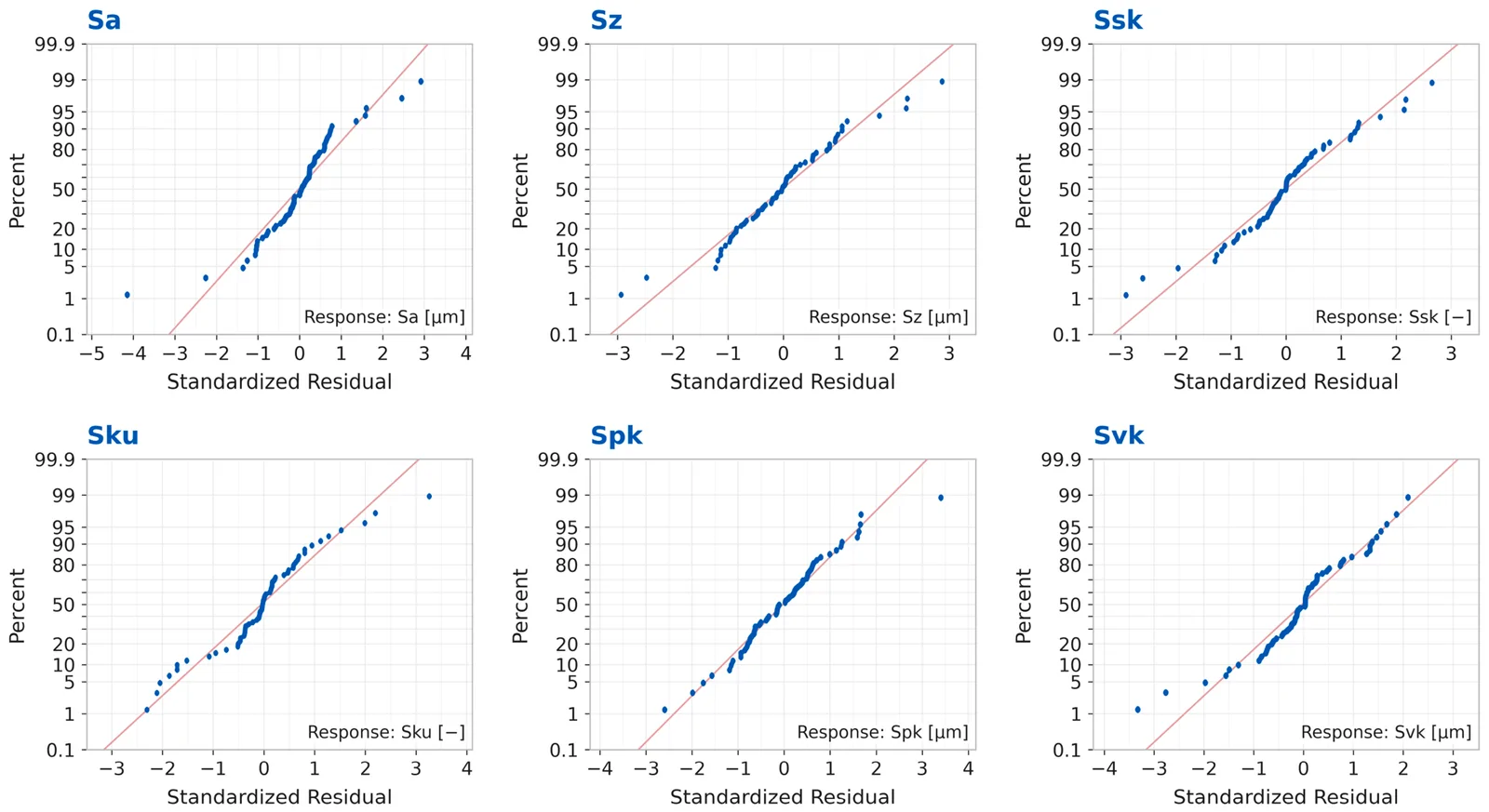
Normal probability plots of standardized residuals for *Sa, Sz, Ssk, Sku, Spk* and *Svk*.

Normal probability plots of standardized residuals (Figure 8) were included for evaluating the normal distribution of residuals (error terms). The residuals closely follow the reference line mainly in the case of the *Sz* and *Spk* parameters, with the presence of few deviations. In other cases, residuals are not as close to the reference line, mainly in the case of *Sku* and *Svk*, which can be related to the specific characteristics of these roughness parameters. Nevertheless, highly pronounced systematic pattern of residuals was not observed at any of the roughness parameters. Hence, it can be stated that the distribution of residuals does not substantially depart from normality for all six cases.

A coefficient of determination (R^2^) was calculated for the interpretation of the proportion of response variance explained by the model. The adjusted coefficient of determination R^2^_adj_, which penalizes the addition of unnecessary independent variables, is also included in Table 4. The highest values were reached in the case of the *Sa* and *Sz* parameters and the lowest in the case of the *Sku* and *Svk* parameters, which is related to the causal character of these measures in relation to the evaluated factors in the form of technological parameters. Nevertheless, the obtained coefficient (R^2^) indicates that the model explains more than approx. 98.42% of the observed variability for the *Sa* parameter in the responses, which is followed by similar values for *Sz*, *Ssk* and *Spk*. For the *Sku* and *Svk* parameters, approx. 13.96% and 13.76% are related to remaining unexplained variation and experimental error, respectively.

The measured spatial texture data were statistically processed using interval diagrams with a 95% confidence level. To identify the degree of individual technological factor influence, a main effects analysis (Main Effects Plots) was applied, where the reference base is the total mean value of the monitored parameters. Interaction plots are not included because all interactions of the parameters were under the level of significance for each evaluated surface parameter.

**Figure 9:**
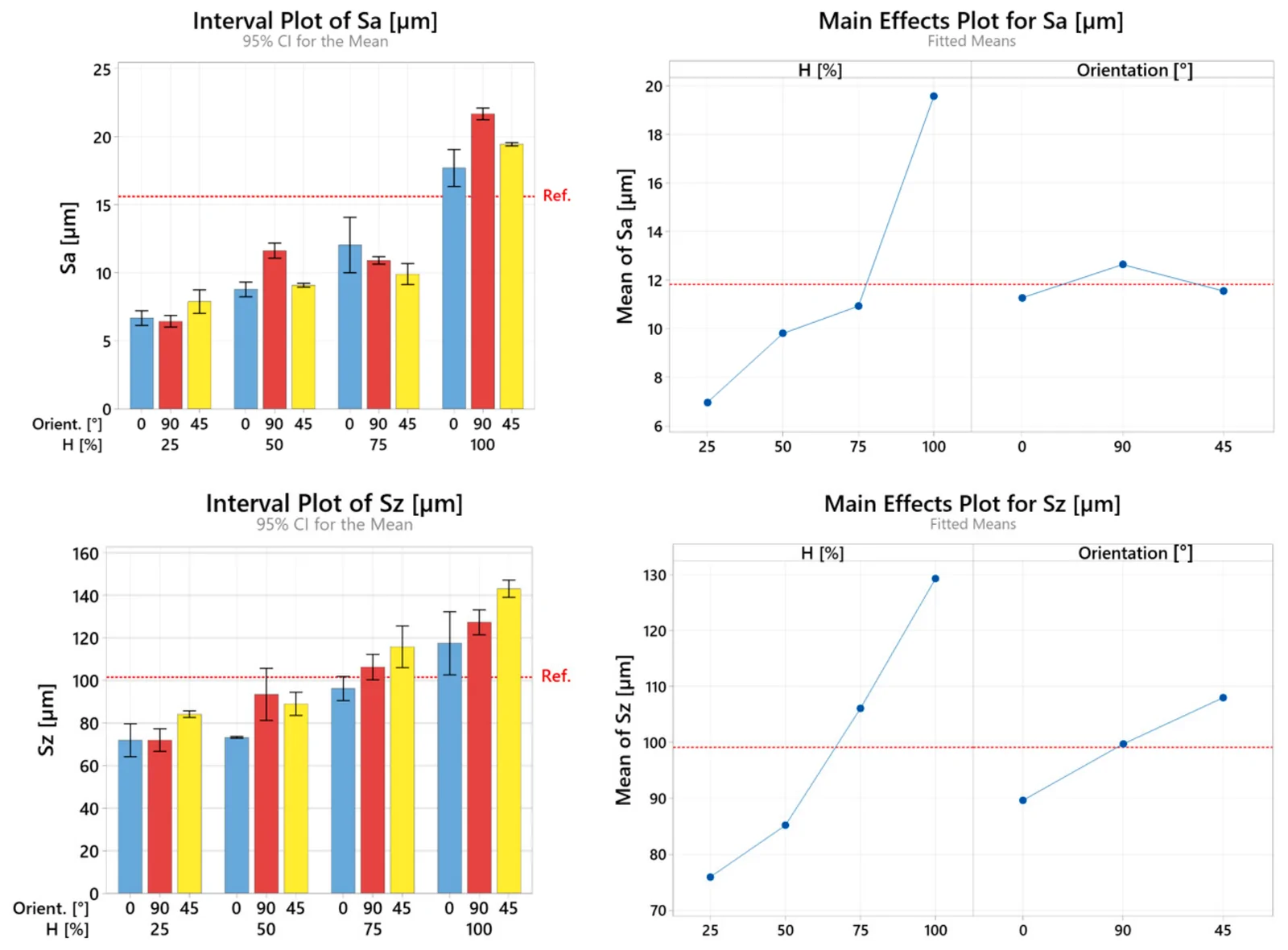
Effect of laser post-processing analysis of main factors with different orientations and *H* on *Sa* and *Sz* parameters (*n* = 5, mean ± SD).

The results for the parameter *Sa* (Figure 9) show a significant reduction in surface roughness at *H* = 25%, 50% and 75%. The maximum smoothing efficiency was recorded with the combination of *H* = 25% and *perpendicular orientation*, where the roughness decreased from *Sa* = 15.6 µm in the reference sample to *Sa* = 6.42 ± 0.31 µm, which represents a 59% reduction. Approximately the same reduction was also observed in [48], where the authors declared that such roughness can positively affect biocompatibility and does not increase the toxic effect on the growth of primary osteoblasts of OB cells in comparison with the ground surface. This progress is a direct consequence of the fine step displacement that ensures uniform exposure of all surface irregularities, which was also proven in [45] while maintaining the same 25% step displacement. Contrariwise, at the setting *H* = 100%, roughness deteriorated in all orientation cases, where the maximum deterioration of 39%, compared to the reference surface at *Sa* = 21.64 ± 0.31 µm, was recorded in the *perpendicular orientation*. This increase confirms the assumption of non-uniform energy distribution in the beam cross-section (Figure 2), where the absence of path overlap (*H* = 100%), in combination with the local beam intensity, generates secondary waviness that exceeds the original roughness of the print [33]. It can be stated that the overlap rate of 75% is a critical threshold for improving the surface quality in terms of surface roughness *Sa*. The main effects analysis shows that the hatching distance *H* is the dominant factor affecting *Sa*, while the polishing orientation shows a more stable character, with a lower degree of influence on this parameter. However, it should be noted that these results were obtained while maintaining a constant area energy density (*ED*), whereas the experimental setup varied the local line energy density (*EL*), which could—in each case—influence the surface homogenization process.

The parameter *Sz*, which is more sensitive to local extremes (protrusions and depressions), shows a different trend compared to that of *Sa*. The reduction of *Sz* was achieved only at *H* = 25% and 50%, with a maximum decrease of 29% (*H* = 25%, *perpendicular orientation*, *Sz* = 71.85 µm). The lower reduction rate of *Sz* compared to *Sa* is attributed to the melt redistribution mechanism. The process of melting peaks and their subsequent flow into adjacent valleys leads to the gradual filling of valleys and cavities [37], which effectively reduces the vertical extent of the texture [33]. In addition, with sufficient overlap (*H* = 25% and 50%), a secondary *HAZ* effect from additional beam passes is noted. Although the extent of the heat-affected zone (*HAZ*) was not been directly investigated, its influence and effect could have been directly altered by changes in the linear energy density (*EL*), which was varied at different heat input levels. This partially remelts the already formed melt, which promotes its homogenization and eliminates extreme height differences. An interesting finding is that *H* = 75% leads to a 5% improvement in *Sz* only for the *parallel* orientation, but for *perpendicular* and *oblique orientations*, *Sz* deteriorates by 5% to 14%. This difference between *Sa* and *Sz* indicates that although the process successfully homogenises the overall surface (melt flow into valleys), at higher values of *H* = 100%, isolated deep grooves of a “gully” character may occur. Nevertheless, the relatively small differences in *Sz* confirm the visual observations via *SEM* analysis of the rounding of the edges of peaks or reliefs due to the influence of *HAZ* (Figure 4). The main effects plot for *Sz*, in contrast to *Sa*, indicates a more significant influence of orientation. This can be attributed to the fact that *parallel orientation* naturally smooths existing layer lines, while *perpendicular* and *oblique orientations* create new, visually distinct relief structures [30], which increase the vertical range of the texture.

**Figure 10:**
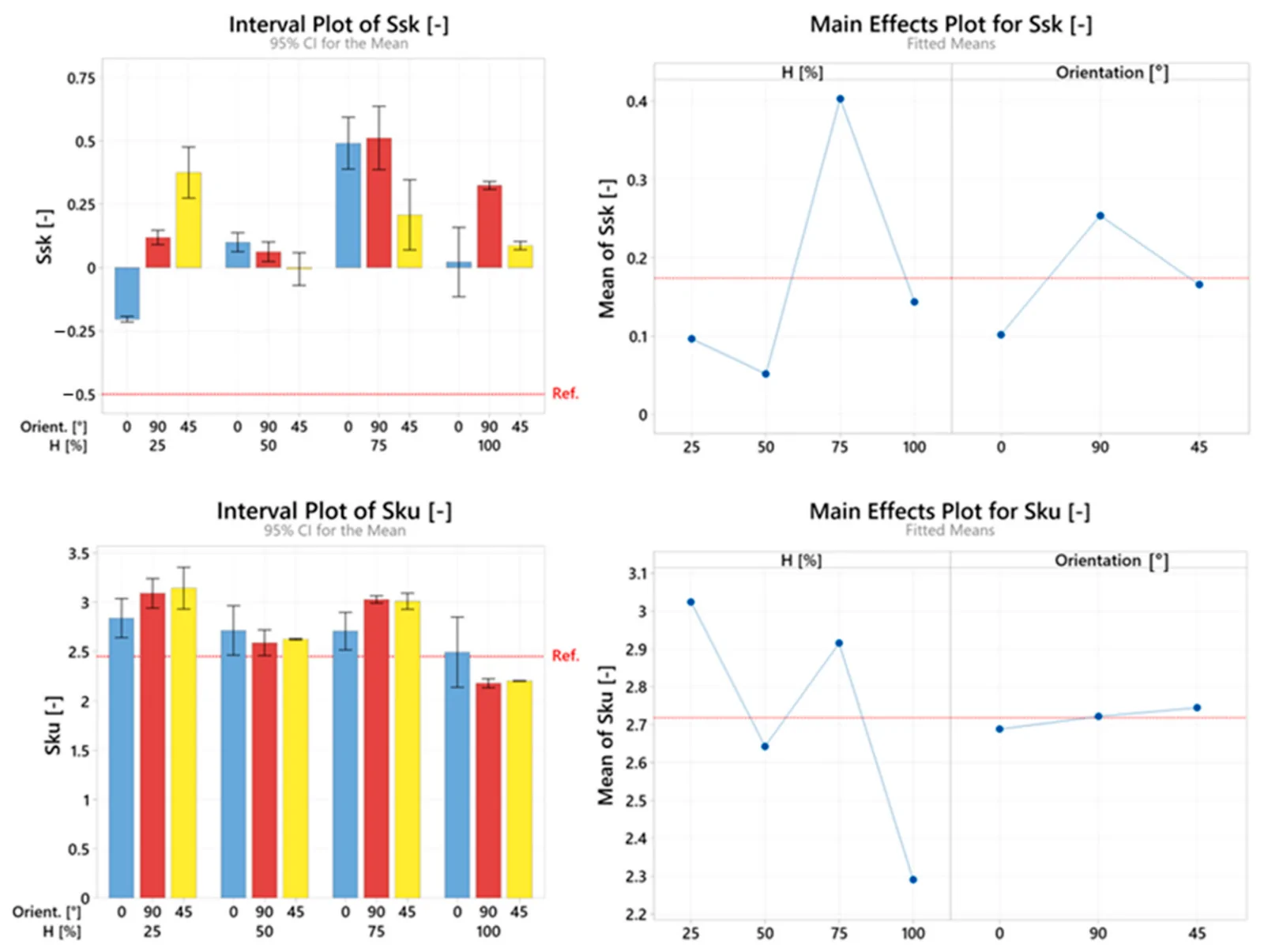
Effect of laser post-processing analysis of main factors with different orientations and *H* on *Ssk* and *Sku* parameters (*n* = 5, mean ± SD).

For a more detailed quantification of topographic changes, the parameters of the distribution of heights, skewness (*Ssk*) and sharpness (*Sku*) were analysed (Figure 10). These parameters provide a more thorough insight into the symmetry of the profile and the morphological character of the peaks.

The reference surface shows a significant negative skewness *Ssk* = −0.5 (*Ssk* < 0), which indicates the predominance of valleys over peaks, and the value of *Sku* = 2.45 (*Sku* < 3) indicates the rounded character of the peaks (platykurtic distribution). This combination is typical for *FDM* technology, where the extrusion of the filament through a circular nozzle naturally creates laterally rounded layers, while the spaces between them (interlayer interfaces) form dominant negative depressions that extend to the places of adhesive connection. However, the negative skewness is typical only for the vertical orientation, since a positive skewness *Ssk* = 0.53 was recorded in the horizontal orientation of the print [33], apparently due to a more thorough filling of the interlayer space, which also proves that the topicality of the post-processing issue is highly relevant in the vertical orientation.

Laser post-processing led to the transformation of the surface to an equilibrium (symmetric) *Ssk* = 0 to a positive *Ssk* (peak dominance). The exception was at *H* = 25% for *parallel orientation*, where the negative character, similar to the raw print, was preserved. This phenomenon can be explained by the dynamics of the process of maintaining a constant areal energy density (*ED*), where a high feed rate was set, in this case (*vs* = 600 mm·s^−1^). Although the total induced energy is identical to the other experimental settings, the extremely short interaction time of the beam with the material was insufficient for complete redistribution of the melt into deep valleys in *parallel orientation*. Another reason may be that the positive skewness and consistent remelting were generated by the high local linear energy density (*EL*) achieved through increasing *H*, and consequently, decreasing *vs*.

In the case of *perpendicular* and *oblique orientations*, this time deficit was eliminated, since these strategies ensure an immediate transition of the beam from the peak to the adjacent valley, which facilitates the melting of the melt and more efficient filling of the interlayer space [30,37]. The most pronounced effect was observed in the *oblique orientation*, where the valleys are filled with material from two adjacent molten peaks, which correlates with the results of the *SEM* analysis.

The *Sku* analysis showed that even after polishing, the surface retained the rounded character of the peaks (*Sku* < 3), which was also observed in [33]. This is most pronounced at *H* = 100%, where the absence of track overlap generates a groove-like morphology. In combination with the thermal influence of the *HAZ*, which affects a wider area than does the beam diameter, regular melting and rounding of potential microrelief peaks occur. The nonlinearity of the technological parameters at *H* = 75% indicates that the irregularity of the step-shift relative to the layer geometry (*H* = 0.315 mm, i.e., 1.5× the layer thickness) induces a chaotic intensity distribution, which leads to unpredictable remelting and local anomalies in the skewness and sharpness of the surface, which is observed in the analysis of the most significant factors.

**Figure 11:**
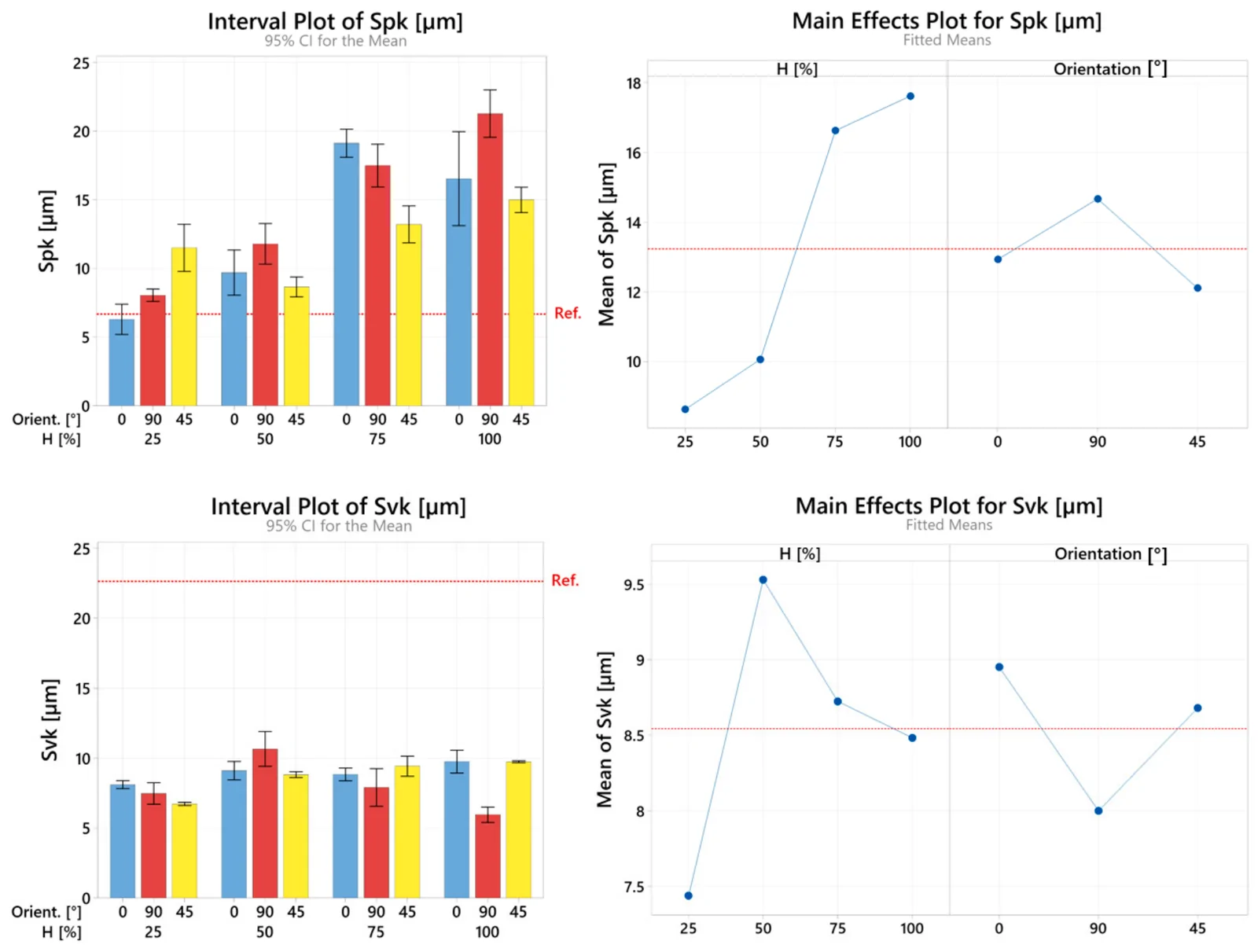
Effect of laser post-processing analysis of main factors with different orientations and *H* on *Spk* and *Svk* parameters (*n* = 5, mean ± SD).

Quantification of the functional characteristics of the surface using the *Spk* and *Svk* parameters provides information about morphological changes in the area of peaks and valleys relative to the core of the surface (Figure 11). Paradoxically, the measured data show that laser post-processing increased the average height of the *Spk* peaks in each configuration.

When compared to the reference sample (*Spk* = 6.67 µm), the most significant increase, up to 219% (*Spk* = 21.28 µm), was recorded at *H* = 100% and *perpendicular orientation*. This increase can be attributed to the formation of new relief structures where (at zero track overlap) the material between the laser tracks remains unaffected, or only partially modified, by the *HAZ* effect. Hence, the resulting topography is formed by new, clearly defined (but rounded) peaks that increase the *Spk* value. The high linear energy density (*EL*), which could have caused more intense local remelting of the material compared to the other *H* settings, may be the cause for such behaviour. Moreover, it is important to note that at *oblique* orientation, these peaks at *H* = 50–100% showed significantly lower values than at *parallel* or *perpendicular orientation*. Again, it confirms the unique character of the *oblique* transition, which, thanks to the bidirectional flow of the melt from adjacent peaks, creates a flatter and more homogeneous morphology.

Contrariwise, the valley depth *Svk* shows a uniform decrease in the range of ~50–70% compared to the reference state after polishing. This trend is a direct consequence of the “filling effect” of the melt at the sharpest interlayer valleys [37]. The redistribution of material into the valleys leads to an increase in the level of the profile centreline and the surface core, which results in a significant reduction in the valley volume below the surface core. The absence of extreme valleys across all orientations confirms that laser polishing successfully homogenises the critical points of the interlayer joints. The analysis of the main effects indicates a mirror trend between *Spk* and *Svk*. This phenomenon is a prerequisite for volume conservation during melting, as in places where the process generates new peak structures (increase in *Spk*), the original valleys or gaps are simultaneously filled (decrease in *Svk*), making the surface more compact and strengthened. However, a comprehensive surface analysis is essential for this confirmation.

**Figure 12:**
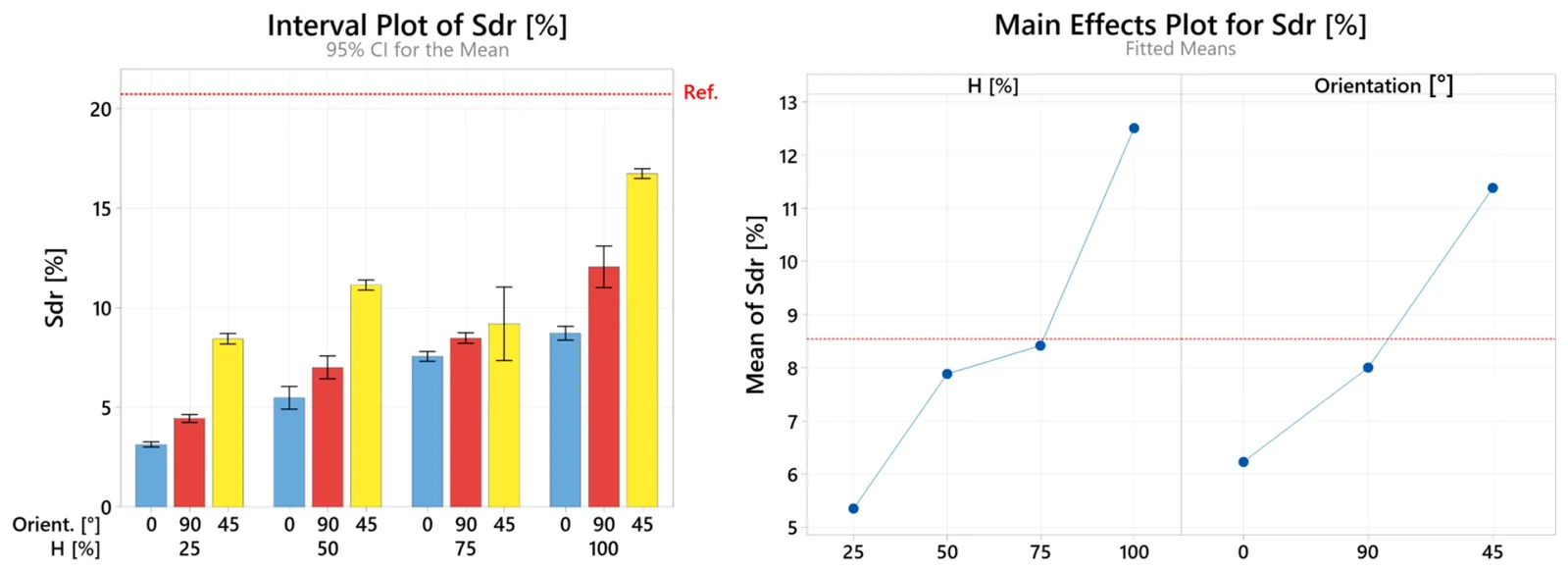
Effect of laser post-processing analysis of main factors with different orientations and *H* on *Sdr* parameter (*n* = 5, mean ± SD).

The *Sdr* parameter (expressing the increment of the developed area) provides a comprehensive view of the degree of homogenization and unification of the modified surfaces (Figure 12). In each tested laser post-processing configuration, a significant decrease in *Sdr* was recorded compared to results for the reference sample (*Sdr* = 20.74%). This trend confirms that the process of melting the layer peaks and their subsequent redistribution into the valleys significantly reduces the overall geometric complexity of the surface. However, the analysis of the most significant factors revealed fundamental differences, depending on the scanning orientation. The highest degree of surface homogenization and unification was achieved by the *parallel* orientation, where *Sdr* increased with increasing *H* = 25–100% to *Sdr* = 3.14%, 5.49%, 7.57%, and 8.73%. The mechanism of smoothing lies in the *parallel orientation* of the beam paths relative to the orientation of the print layers, where the melt is carried in an identical direction, which prevents the fragmentation of the surface into the secondary microreliefs observed via optical analysis and *SEM*. In contrast, the *perpendicular orientation* (*Sdr* = 4.45%, 7.02%, 8.49% and 12.06%) and the *oblique* orientation (*Sdr* = 8.45%, 11.15%, 9.20% and 16.75%) show higher values of the developed area compared to those for the *parallel* orientation. This increase is a consequence of the geometric distribution of the surface into several distinct structures. With *perpendicular* or *oblique* movement of the beam, the originally continuous lines of the layers are divided into discrete relief structures. These newly formed micro-morphological elements (although rounded) increase the total texture area and thus, the *Sdr* parameter. It can be stated that while *parallel* orientation tends towards maximum macroscopic smoothness, *oblique orientation* creates a more complex, functionally structured morphology, which is also confirmed by mirror trends in the functional height parameters.

### 3.4. Mechanical Properties

The tensile-test results are reported as peak force (the maximum recorded load), peak stress (the engineering tensile stress corresponding to that load), and elongation at peak force (the recorded extension at maximum load). The tests were performed in accordance with ISO 527-2:2012 [43], as described in Section 2.3, and the results are summarized in Table 4.

**Figure 13:**
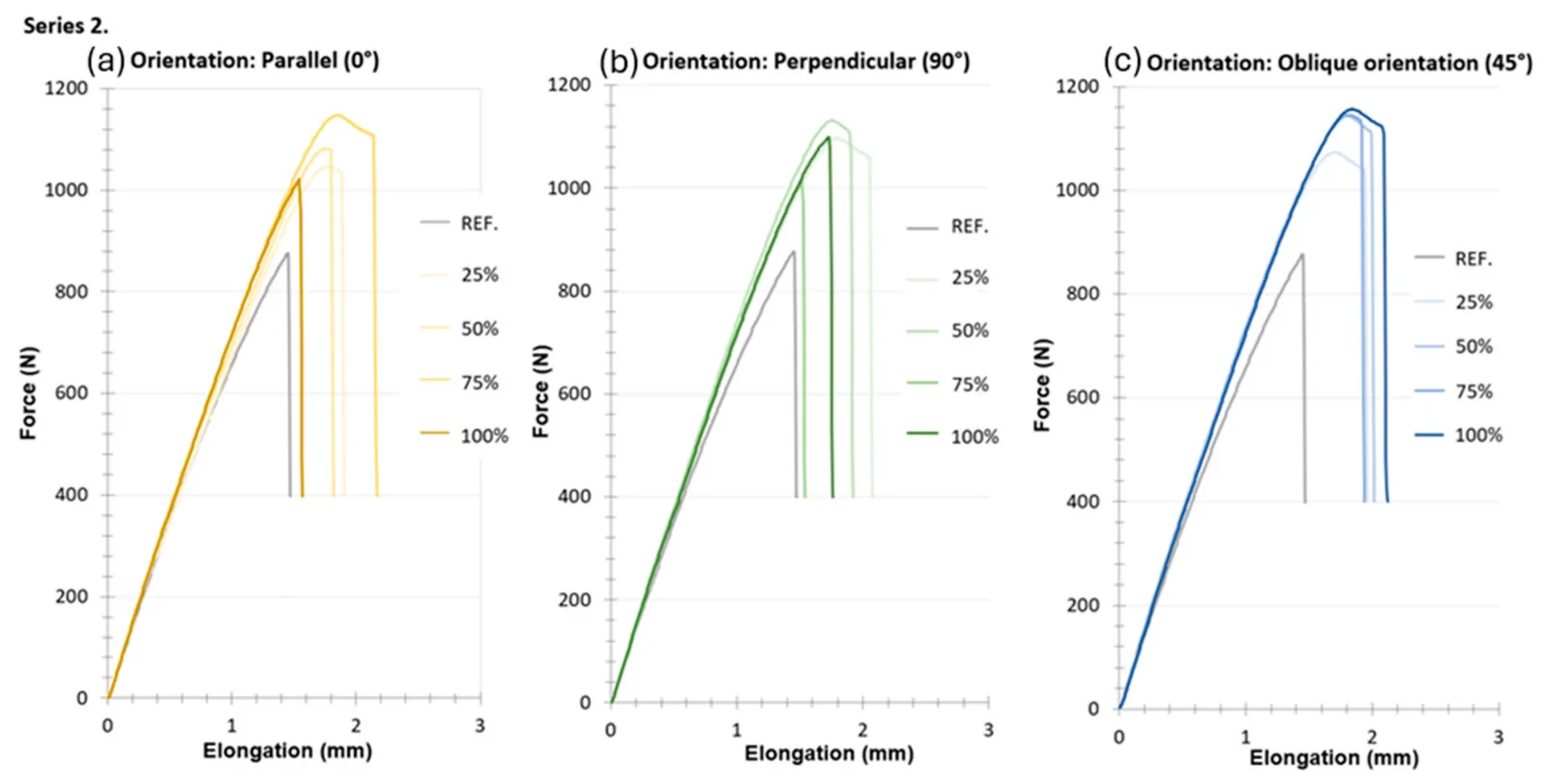
Representative tensile test graphs (force–elongation) for sample series 2 at different laser scanning orientations: (**a**) *parallel orientation* (0°), (**b**) *perpendicular orientation* (90°), and (**c**) *oblique orientation* (45°). A reference sample (*REF*) is included for comparison in each diagram.

The results of the static tensile test (Table 5 and Figure 13) demonstrated a positive effect of laser treatment on the strength characteristics of the *PLA* material in almost all tested configurations. Subsequently, with the assumptions from the previous analyses, the effect of polishing on the samples can be determined in two ways:

1. *Strengthening effect*: Increased structural integrity due to the melting of the layer peaks and subsequent redistribution of the melt into the interlayer valleys, without degradation of the *PLA* material.
2. *Degradation effect*: For specific combinations of parameters, a negative effect of local thermal overload was observed, associated with the formation of secondary microporosity or undesirable relief structures, which limited the effective cross-section of the modified zone.

The reference samples achieved an average maximum force of 1050.46 N, a peak engineering stress of 26.26 MPa, and a crosshead displacement at peak force of 1.67 mm. In most modified states, the measured mechanical response was comparable to or higher than that of the reference specimens. The highest values were obtained for the *oblique* orientation (45°), particularly at *H* = 75% and 100%. At *H* = 100%, this configuration achieved the highest average maximum force of 1168.33 N, the highest peak engineering stress of 29.21 MPa, and a crosshead displacement at peak force of 1.86 mm. Compared with the reference state, these values represent increases of approximately 11.2% in maximum force, 11.2% in peak engineering stress, and 11.4% in crosshead displacement at peak force.

A similar trend was observed for the *oblique* orientation (45°) at *H* = 75%, where the maximum force reached 1154.83 N, and the peak engineering stress reached 28.87 MPa. These results are consistent with the favourable mechanical response observed for the oblique scanning strategy. The surface observations suggest that melt redistribution may contribute to this behaviour; however, the present data do not directly demonstrate a specific strengthening mechanism or improved interlayer bonding.

A different trend was observed for the *parallel* orientation (0°), which showed the largest variation in mechanical response among the tested conditions. At *H* = 25%, the values were practically comparable to the reference state, with a peak engineering stress of 26.23 MPa. At *H* = 50%, however, the maximum force decreased to 871.53 N, the peak engineering stress to 21.79 MPa, and the crosshead displacement at peak force to 1.51 mm. Compared with the reference state, this corresponds to a decrease of approximately 17.0% in maximum force and 9.6% in crosshead displacement at peak force. At *H* = 75%, the peak engineering stress increased to 28.70 MPa, and the crosshead displacement at peak force reached 1.86 mm. The pronounced decrease at *H* = 50% may reflect an interaction between the laser path spacing and the printed-layer relief. The programmed spacing of 0.210 mm is close to the nominal layer height of 0.20 mm, so adjacent parallel paths could interact with similar positions relative to the interlayer peaks and valleys. This explanation remains tentative: *H* = 100% is also close to twice the layer height, while scanning speed and line energy varied with *H*. The available surface and fracture images cannot distinguish these possible contributions or establish whether subsurface defects play a role.

The *perpendicular* orientation (90°) showed a more stable mechanical response than did the parallel orientation. The highest values within this group were obtained at *H* = 50%, where the maximum force reached 1132.66 N, the peak engineering stress was 28.32 MPa, and the crosshead displacement at peak force equalled 1.77 mm. At *H* = 25%, the peak engineering stress was 27.41 MPa, and the crosshead displacement at peak force was 1.81 mm. At *H* = 75% and 100%, the peak engineering stress decreased to 27.21 and 27.10 MPa, respectively. These differences may be associated with changes in the laser-induced surface morphology and thermal conditions; however, the available results do not provide direct evidence of the mechanism responsible for the observed mechanical response.

It should also be noted that, just as with surface roughness, the results regarding mechanical properties cannot be unequivocally attributed solely to the isolated effects of *H* associated with the change in orientation. An undefined thermal shock—resulting from the change in local linear energy density (*EL*) that accompanied the variation of *H*—may also have played a role in the process.

Representative tensile test graphs (selected from series 2) confirm the typical behaviour of *FDM-PLA* material with brittle to quasi-brittle failure. The curves show an almost linear elastic region, followed the tensile strength limit being reached and a sharp decrease in force, without significant plastic deformation. This can be defined as a typical phenomenon of interlayer adhesion, which is significantly influenced by the orientation of the print build-up [49]. Hence, the adhesion of the interlayers is influenced by the intensity of the melt pool in its surface and subsurface regions after laser post-processing [31,32]. In the *parallel orientation* (0°), the differences between the curves were most pronounced. The lowest load capacity was shown by the configuration *H* = 50%, while *H* = 75% achieved higher force and elongation levels. The *perpendicular orientation* (90°) showed a more stable course, with the highest load level recorded at *H* = 50%. The most favourable behaviour was observed at the *oblique orientation* (45°), especially at *H* = 75% and *H* = 100%, where the curves were shifted towards both higher force and greater elongation. This trend confirms that *oblique* scanning promotes more efficient filling of interlayer valleys and more uniform melt redistribution.

Based on the results, it can be stated that the best mechanical properties were not achieved in the state with the lowest surface roughness, but in the state in which the most favourable compromise between a homogeneous surface and the preservation of the structural continuity of the modified layer occurred. Hence, the mechanical response was not controlled only by the degree of track overlap, but primarily by the interaction between the scanning orientation, the hatch distance and the geometry of the original layered structure. From this point of view, the 45° *oblique orientation* appears to be the most suitable strategy.

#### Statistical Analysis of Mechanical Response

Peak force (Force Peak, N) was selected as the primary response variable for the statistical evaluation of the mechanical response. A two-factor general linear model (GLM) was fitted to the data obtained from the laser-modified specimens, with orientation (°) and hatch distance *H* (%) treated as fixed factors. The model included the main effects of both factors and their interaction. Five replicate measurements were analysed for each combination of orientation and hatch distance (*n* = 5). The reference condition was excluded from the two-factor model because neither a scan orientation level nor a hatch distance value could be assigned to it. Since Stress Peak was calculated from Force Peak using the same nominal cross-sectional area for all specimens, it represents a linear rescaling of Force Peak. Therefore, no separate inferential analysis of peak stress was performed, as it would yield the same statistical conclusions as the analysis of peak force. Statistical significance was assessed at α = 0.05. The assumptions underlying the GLM were evaluated using residual diagnostics. Residual normality was assessed using the Anderson–Darling test, while homogeneity of variance was assessed using Levene’s test. The residuals were also examined graphically using a normal probability plot and a plot of residuals versus fitted values.

The GLM results revealed statistically significant effects of orientation, hatch distance, and their interaction on Force Peak. The two-way analysis of variance (ANOVA) showed that both factors and their interaction significantly affected Force Peak. The orientation factor represents the three laser scanning directions examined: *parallel*, *perpendicular*, and *oblique*. Its effect on Force Peak was statistically significant, with F(2, 48) = 136.09, *p* < 0.001, indicating that mean Force Peak differed significantly among the scanning orientations. The hatch distance factor represents the four investigated levels: *H* = 25, 50, 75, and 100%. Hatch distance also had a statistically significant effect on Force Peak, with F(3, 48) = 76.57, *p* < 0.001. However, given the significant interaction between the two factors, these main effects must be interpreted in the context of their interaction. The orientation × hatch distance interaction was also statistically significant, with F(6, 48) = 77.76, *p* < 0.001. This significant interaction indicates that the effect of hatch distance on Force Peak differed among the scanning orientations. Thus, changes in hatch distance produced different changes in Force Peak, depending on the scanning orientation used. Accordingly, interpreting the effects of orientation and hatch distance in isolation would be inappropriate, as the mechanical response depends on their combination. The model achieved R^2^ = 95.28%, indicating that orientation, hatch distance, and their interaction explained 95.28% of the variability in Force Peak. The adjusted R^2^ was 94.20%, and the predicted R^2^ was 92.62%. The residual standard deviation of the model was S = 24.17 N. Thus, the high R^2^ indicates that the fitted model explained a large proportion of the variability in Force Peak. The assumptions of the two-factor GLM were subsequently assessed through residual analysis. The Anderson–Darling test did not indicate a statistically significant departure of the residuals from a normal distribution (AD = 0.593, *p* = 0.119). Since *p* > 0.05, the assumption of residual normality was not rejected. Likewise, Levene’s test did not reveal statistically significant differences in variance among the combinations of orientation and hatch distance (*p* = 0.661). Therefore, at α = 0.05, no substantial violations of the assumptions of residual normality or homogeneity of variance were detected for the fitted GLM.

The significant orientation × hatch distance interaction is also evident from the mean Force Peak values reported in Table 5. For the *parallel* orientation, Force Peak varied markedly with hatch distance, with the lowest mean value of 871.53 N recorded at *H* = 50%, whereas it reached 1148.07 N at *H* = 75%. For the *perpendicular* orientation, the response was more stable, with the highest mean Force Peak of 1132.66 N recorded at *H* = 50%. For the *oblique* orientation, the highest mean Force Peak values were observed at larger hatch distances, with a maximum of 1168.33 N attained at *H* = 100%. These contrasting trends confirm that the effect of hatch distance on Force Peak depended on orientation.

**Figure 14:**
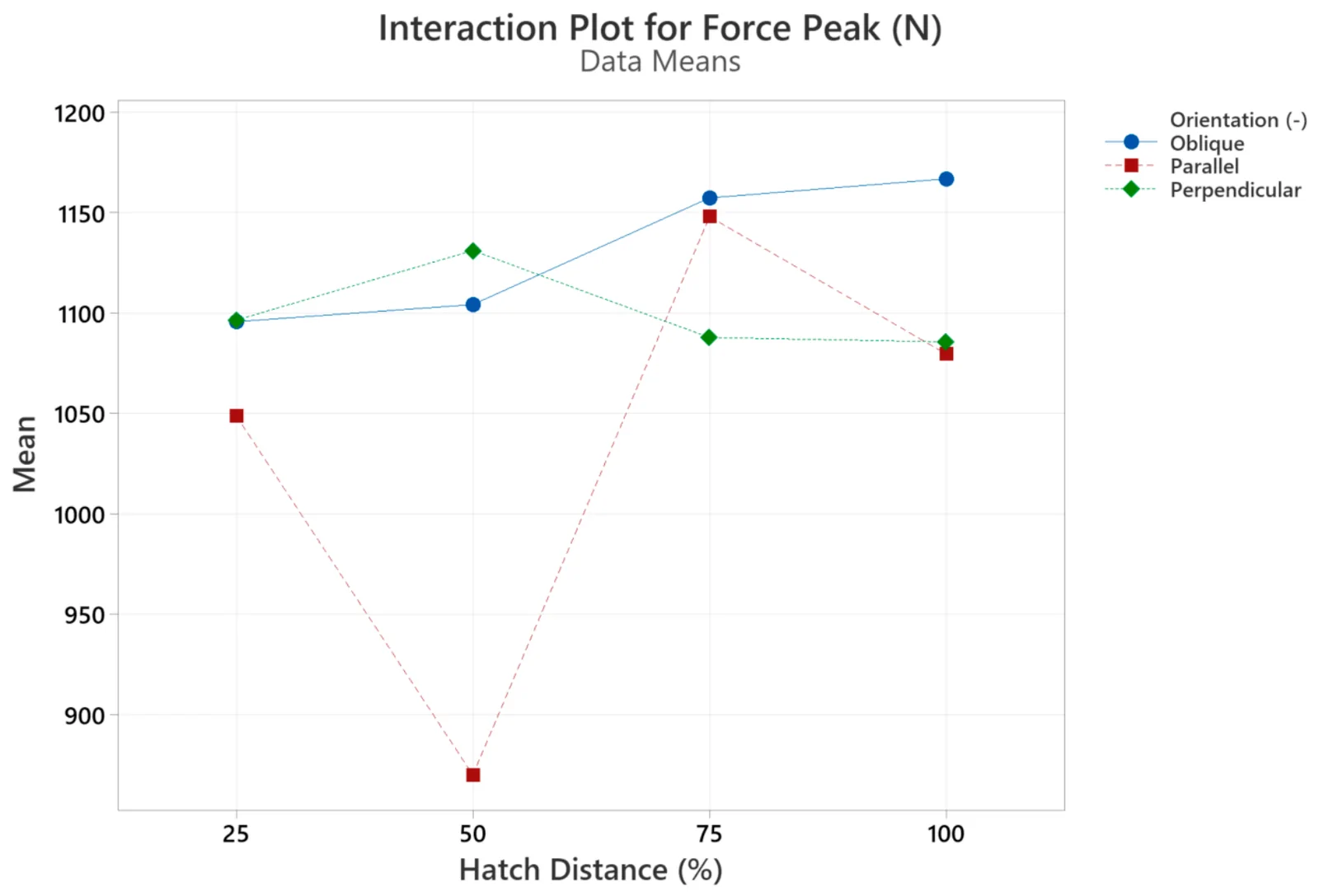
Interaction plot showing the effect of orientation and hatch distance on Force Peak.

The interaction plot in Figure 14 shows that the effect of hatch distance on Force Peak varies markedly with orientation. For the *oblique* orientation, Force Peak generally increases with increasing hatch distance, with the highest mean value observed at *H* = 100%. For the *parallel* orientation, the response is markedly nonlinear: Force Peak decreases substantially at *H* = 50%, followed by a sharp increase at *H* = 75%. For the *perpendicular* orientation, the changes are less pronounced, with the maximum observed at *H* = 50%.

The distinctly different trends across orientations provide visual support for the statistically significant orientation × hatch distance interaction identified in the GLM analysis, with F(6, 48) = 77.76, *p* < 0.001. This indicates that the effect of hatch distance on Force Peak depends on the scanning orientation. Therefore, these two factors must be considered jointly when interpreting the mechanical response.

**Figure 15:**
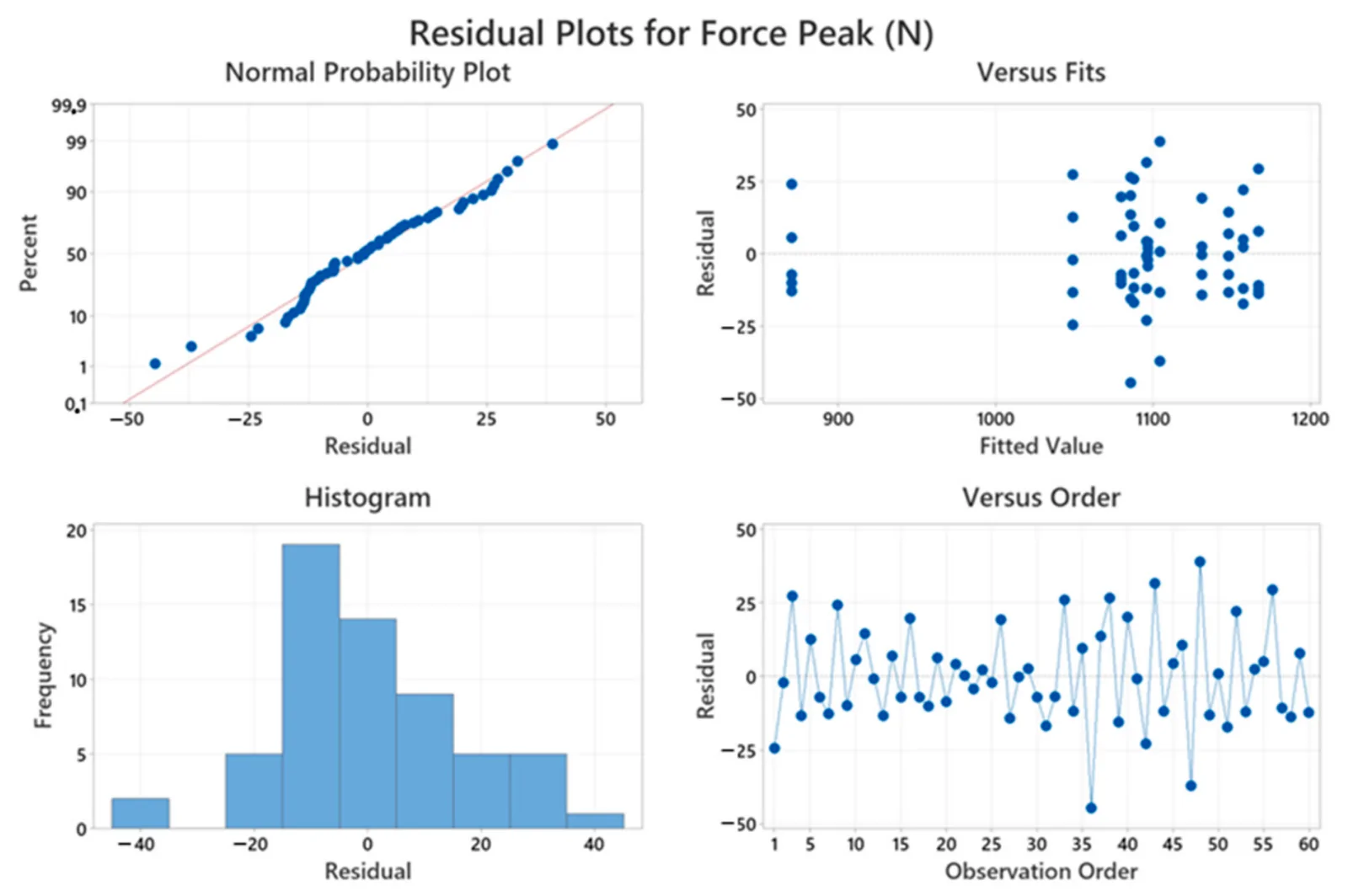
Residual diagnostic plots for the two-factor GLM of Force Peak, including the normal probability plot, residuals versus fitted values, histogram of residuals, and residuals versus observation order.

Graphical residual diagnostics (Figure 15) revealed no clear systematic pattern in the residuals with respect to either the fitted values or the observation order. Moreover, the Anderson–Darling test did not indicate any statistically significant departure of the residuals from a normal distribution.

### 3.5. Fractographic Analysis of Tensile Fracture Surfaces

**Figure 16:**
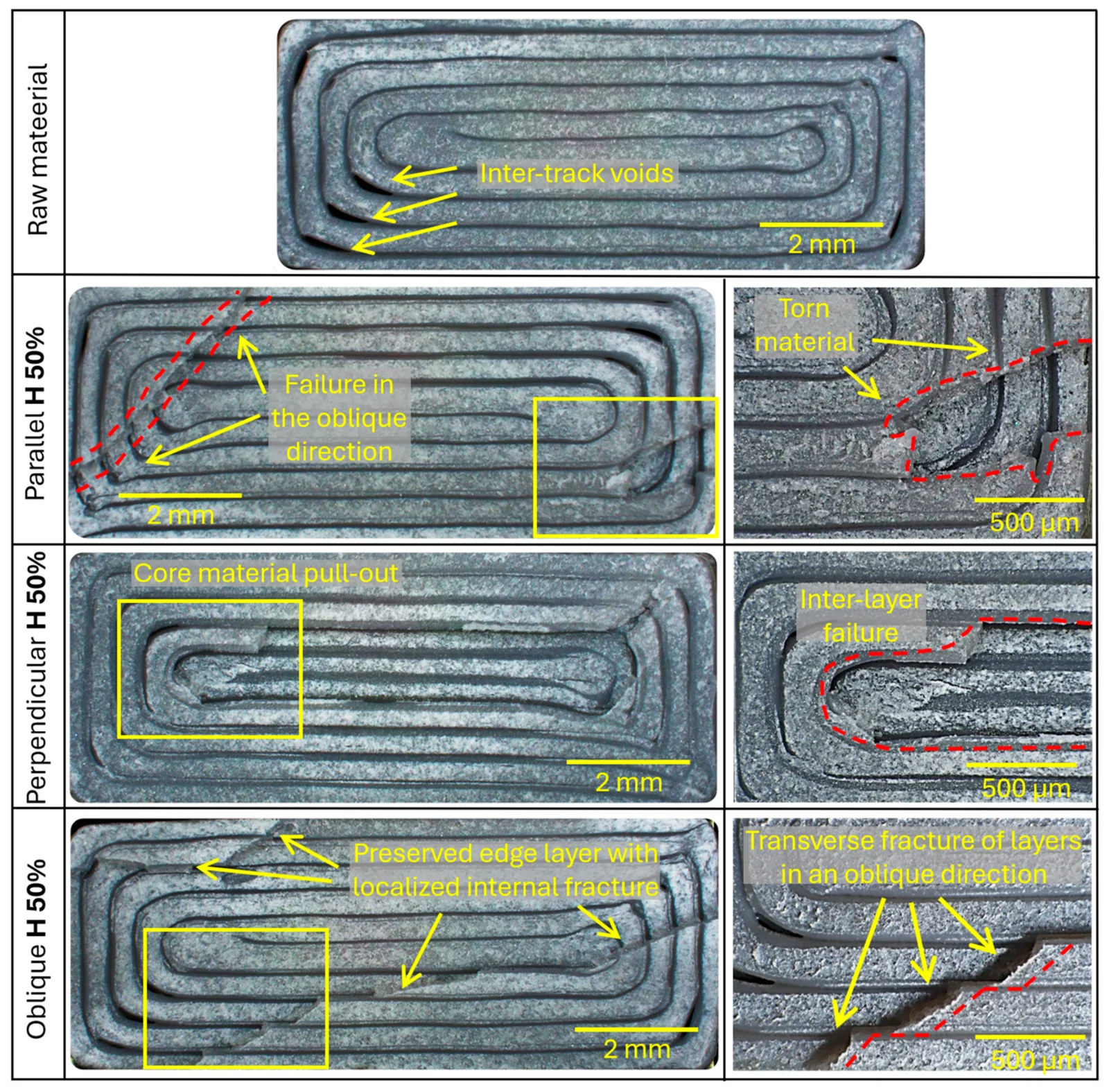
Effect of laser post-processing with different orientations on the fractural characteristic (optical analysis of sample cross-sections after tensile testing).

To comprehensively assess the impact of laser modification on structural integrity, fractographic analysis of fractured surfaces after static tensile testing was performed. The fracture behaviour of the reference sample was compared with the modified samples for each scanning strategy (Figure 16). For each scanning orientation, one specimen processed at *H* = 50% was selected for comparison with the untreated reference. The fractographs provide a descriptive comparison of fracture morphology but do not establish crack-initiation sites nor the mechanisms underlying the results obtained at other hatch distances. The fracture behaviour of the as-printed sample shows signs of a typical brittle interlayer failure, without the presence of plastic deformation, fibre pulling or delamination of individual lines. This clean fracture is a consequence of the low interlayer adhesion, which is the primary limiting factor of *FDM* vertical printing technology. Considering the visual analysis, it is clear that the integrity of the interface was negatively affected by internal porosity (inter-track voids), observed mainly in the areas of fibre rounding that are created during the extrusion process [30,31].

In the case of *parallel orientation* (0°), a complex three-layer failure can be observed. While the top layer exhibits an oblique fracture orientation with respect to the layer orientation, the lower layers are dominated by a local material pull-out mechanism. This condition was also observed in [31] and may be associated with melt redistribution into the interlayer valleys, followed by additional remelting in the heat-affected zone of adjacent laser passes, potentially producing a more continuous surface.

In the case of *perpendicular orientation* (90°), the fracture is characterised by the tearing out of the central segments of the sample, with simultaneous failure in two layers. Although the segmentation of the surface into microreliefs (confirmed by optical analysis) reduced the overall compactness compared to that of the *parallel orientation*, the observed fracture pattern may be consistent with improved subsurface adhesion and a shift of failure toward the less-heat-affected interior of the sample; however, the available images do not confirm this mechanism nor identify the site of fracture initiation.

Fractography of the *oblique orientation* (45°) showed partially preserved peripheral layers and localized internal fracture. Compared with the predominantly interlayer fracture of the untreated specimen, the fracture path extended across multiple deposited layers and included transverse fracture of individual filaments [30,31]. These features are consistent with the more continuous surface morphology observed for this scanning strategy. The largest mechanical improvement was measured at *H* = 100%, where peak force and calculated peak engineering stress were both 11.2% higher than in the reference specimens. However, the post-fracture images at *H* = 50% do not establish the site of crack initiation nor demonstrate the mechanism responsible for the improvement measured at *H* = 100%.

The results indicate that scan orientation and hatch distance (*H*) should be selected according to the required surface and mechanical properties of the laser-treated *FDM*-printed *PLA* components [32]. Analysis of these data may help guide process selection [50] and provide input for future predictive models, including machine-learning approaches [41]. The preferred processing condition depended on the response being evaluated. The lowest observed mean *Sa* was obtained with *perpendicular* scanning at *H* = 25%, whereas *parallel* scanning at *H* = 25% produced the lowest *Sdr*. In contrast, *oblique* scanning at *H* = 100% yielded the highest mean peak force and peak stress. Thus, none of the tested conditions can be designated as a general optimum for both surface topography and tensile performance. The processing strategy should be selected according to the required property, while recognising the trade-offs between mean roughness, developed surface area and mechanical response. For the investigated surface topography, the smaller programmed hatch distances of *H* = 25% and 50% (0.105 and 0.210 mm) generally produced more favourable smoothing results. However, the trends depended on the parameter and scan orientation: *Sa* was also reduced under some conditions at *H* = 75%, and *Svk* and *Sdr* did not deteriorate uniformly at larger hatch distances. The obtained data thus suggest that a printed layer thickness of 0.20 mm could represent a critical threshold for the distance between paths (specifically regarding mechanical properties), though this hypothesis cannot be independently verified based on these results. Previous studies have also reported successful smoothing with relatively small hatch spacings [29,35,39], but their results do not establish a universal threshold applicable to the present material and processing conditions. Further experiments at intermediate hatch distances, fixed scanning speeds and different layer thicknesses are needed to examine this possible relationship. Consistent with previous observations that laser processing modifies the layer-induced relief of vertically printed *PLA* [37], the present results show that scan orientation influences the resulting surface morphology. *Parallel* scanning at H = 25% produced the lowest *Sdr*, whereas distinct relief features were observed for some *perpendicular* and *oblique* scanning conditions at larger tested hatch distances [33,42]. Some studies have shown that the creation of structures can have a potentially positive effect on wettability [39], thereby expanding their range of applications [48]. However, further research in this area is essential for specific practical applications [48]. The highest mean peak force was obtained with oblique scanning at *H* = 100%, exceeding the reference value by 11.2%. This mechanical improvement does not, by itself, demonstrate that the resulting surface was the most homogeneous. However, it should be noted that these findings cannot be isolated and attributed solely to the change in parameter *H* while maintaining the area energy density *ED*. In the presented manuscript, the change in *H* was compensated by a change in the scanning speed *vs*, thereby altering the local linear energy density *EL*. This could have significantly contributed to the differing response of the local material melting and the resulting surface. Therefore, these results should be interpreted strictly as findings obtained while maintaining the area energy density *ED*, rather than as results obtained through the systematic maintenance of linear energy density.

## 4. Conclusions

The presented study provides a comprehensive view of the influence of laser polishing strategy (*parallel* 0°, *perpendicular* 90° and *oblique* 45°) and hatch distance parameter ***H*** (25%, 50%, 75% and 100%) on the topographical integrity and mechanical response of vertically oriented *FDM*-*PLA* prints. The conditions that produced the lowest roughness differed from those that yielded the highest tensile performance. The main findings are summarized as follows:

1. Scan orientation influenced the resulting surface relief. Parallel scanning markedly reduced the developed surface area, perpendicular scanning produced a hatch-dependent microrelief, and oblique scanning yielded a more homogeneous surface appearance, accompanied by a shift towards positive profile skewness.
2. The surface morphology showed a porous character, created by gas leakage from the lower areas of the interlayer joint, or expansion of partially degraded material in the subsurface layers, created by insufficient heat removal from the melt pool location.
3. The maximum reduction of *Sa* roughness by 59% was recorded in the perpendicular orientation with *H* = 25%, while *Sz* was reduced by a maximum of only 29% under the same conditions. This difference shows that a substantial improvement in mean roughness does not necessarily produce an equivalent reduction in maximum surface height. Both parameters should therefore be considered when selecting laser polishing conditions for components with specific surface quality requirements.
4. Fractography analysis indicated the improvement in surface adhesion, as a multilayer failure was present in each case after laser post-processing, and at the same time, the tearing of the internal fibres occurred, in which the peripheral fibres remained intact.
5. The most significant mechanical improvement was identified at the *oblique* orientation (45°), where an increase in the maximum force by 11.2% compared to the ref. sample value was observed. This is attributed to the effective flooding of the valleys from two adjacent peaks. Conversely, with an unfavourable geometry (identical *parallel* orientation) and a track spacing symmetry of 50%, the maximum force can deteriorate by 17% due to local valley deepening and stress concentration.
6. Even with scanning speed *vs* compensation for variations in *H* aimed at achieving a uniform area energy density (*ED*); changes in linear energy density (*EL*) can still affect the process, potentially influencing local melting and material response.

Together, these findings provide a basis for selecting the scan orientation and hatch distance according to the surface and mechanical requirements of vertically printed *PLA* components. Future work should investigate the origin of the observed cavities, the influence of cooling conditions, and the applicability of this approach to composite filaments.

## Figures and Tables

**Table 1:** Overview of studies on laser post-processing of *FDM* prints.

Study (Year)	3D Printing Parameters				Laser Polishing Parameters				Main Results
	Fila-ment	Printing Temperature [°C]	Layer Thickness [mm]	Printing Speed [mm·s^−1^]	Type	Power [W]	Hatch Distance [mm]	Scan Speed [mm·s^−1^]	
[28] 2017	*PLA*	-	0.3	-	CO_2_	0.75–3.75	0.152	43–180	Increasing *ED* reduced surface roughness by up to 97%.
[29] 2018	*PLA/ABS*	-	0.2	-	CO_2_	3, 4	0.025–0.2	50, 100, 150	Optimal power is required to maintain a shallow-melt regime and avoid over-melting.
[30] 2019	*PLA*	220	0.2	16.67	FL	3, 4	0.04	200	Enhanced heat distribution via larger beam diameter improves polishing quality.
[31] 2019	*Al-PLA*	200	0.2	16.67	FL	4, 5	0.04	100	Polishing strengthens fibre-matrix connection and stress distribution.
[32] 2020	*CU-PLA*	-	0.2	13.33	FL	4, 5	0.04	150	Additional melting and defect removal reduce delamination risk.
[33] 2020	*Al-PLA*	200	0.2	16.67	FL	2	-	50, 100, 150, 200	Low feed rates cause excessive energy density, leading to material spattering and surface deterioration.
[34] 2021	*PLA*	-	0.1	58	FL	5	0.035	1200	Excessive energy concentration leads to evaporation and mass reduction.
[35] 2023	*PLA*	160	0.26	-	CO_2_	4.2	0.1–0.18	200	Heating efficiency and material response governed by focal length and hatch distance.
[36] 2023	*PLA*	200	0.05–0.3	40	FL	3–30	0.01	50–500	Material colour and absorption spectrum dictate the resulting thermal response.
[37] 2024	*PLA*	210	0.2	50	FL	1–16		600–1000	Improved surface smoothness achieved with lower infill density.
[38] 2025	*ASA*	265	0.4	40	CO_2_	7–11	0.05	150–200	Increased hardness and reduced roughness via gravitational melt flow.
[39] 2025	*PETG*	210	0.8	50	FL	1–4	0.01–0.12	300–900	Control of surface wettability (hydrophobic to hydrophilic) via post-processing parameters.
[40] 2025	*ABS* *Nylon*	-	-	50–150	CO_2_	6–12	0.15–0.25	50–150	Wet processing reduced roughness by 21% compared to dry processing.
[41] 2026	*PLA*	220	0.2	-	CO_2_	10–30	-	38–251	The ability to use machine learning for the reliable prediction of laser polishing roughness.

**Table 2:** Printing parameters, geometry and quality of samples.

**Printing parameters (Bambu Lab X1 Carbon (X1C))**						
Layer thickness	Nozzle diameter	Nozzle temperature	Bed temperature	Cooling	Infill density	Build orientation
0.2 mm	0.4 mm	205 °C	60 °C	Active fan	100%	z-axis
	**Geometry of samples (ISO 527-2:2012, type 1B [43])**					
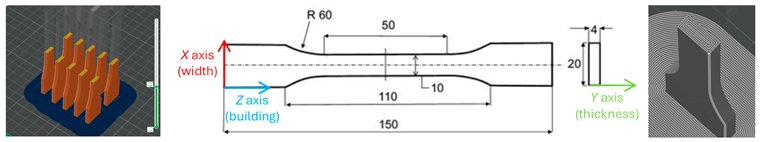						
**Quality of samples**						
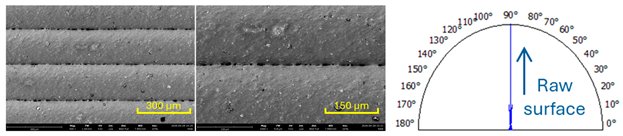						
**Roughness of samples (*N* = 5)**						
*Sa* [µm]	*Sz* [µm]	*Ssk* [-]	*Sku* [-]	*Spk* [µm]	*Svk* [µm]	*Sdr* [%]
15.60 ± 0.52	101.60 ± 12.46	−0.50 ± 0.07	2.45 ± 0.07	6.67 ± 1.51	22.64 ± 1.22	20.74 ± 0.74

**Table 3:** Technological parameters for the experiment.

*P* [W]	*WD* [mm]	*GW* [mm]	*H* [%]	*H* [mm]	*vs* [mm·s^−1^] (% From Nominal)	*EL* [J·cm^−1^]	*ED* [J·cm^−2^]	*Orientation*	Rep. [-]
10	20	0.42	25	0.105	600 (400%)	0.167	15.9	*Parallel* (0°) *Perpendicular* (90°) *Oblique* (45°)	5×
			50	0.21	300 (200%)	0.333			
			75	0.315	200 (133%)	0.500			
			100	0.42	150 (100%)	0.667			

**Table 4:** Calculated coefficients R^2^ and R^2^_adj_ for the *Sa*, *Sz*, *Ssk*, *Sku*, *Spk* and *Svk* parameters.

**Parameter**	*Sa*	*Sz*	*Ssk*	*Sku*	*Spk*	*Svk*
R^2^ [%]	98.42	94.18	92.18	86.04	93.91	86.33
R^2^_adj_ [%]	98.06	92.85	90.39	82.84	92.52	83.19

**Table 5:** Mechanical properties of vertically layered *FDM-PLA* samples after laser post-processing.

Orientation	Hatch Distance [%]	Force Peak [N]	Stress Peak [MPa]	Elongation Peak [mm]
Reference	-	1050.46 ± 8.53	26.26 ± 0.21	1.67 ± 0.19
*Parallel* (0°)	25	1049.08 ± 25.96	26.23 ± 0.65	1.74 ± 0.03
	50	871.53 ± 19.91	21.79 ± 0.50	1.51 ± 0.23
	75	1148.07 ± 13.94	28.70 ± 0.35	1.86 ± 0.02
	100	1080.39 ± 16.45	27.01 ± 0.41	1.65 ± 0.10
*Perpendicular* (90°)	25	1096.42 ± 4.21	27.41 ± 0.11	1.81 ± 0.02
	50	1132.66 ± 16.71	28.32 ± 0.42	1.77 ± 0.03
	75	1088.57 ± 22.32	27.21 ± 0.56	1.60 ± 0.09
	100	1084.10 ± 37.84	27.10 ± 0.95	1.71 ± 0.04
*Oblique* (45°)	25	1098.23 ± 27.36	27.46 ± 0.68	1.75 ± 0.04
	50	1108.31 ± 38.30	27.71 ± 0.96	1.75 ± 0.08
	75	1154.83 ± 21.36	28.87 ± 0.53	1.82 ± 0.03
	100	1168.33 ± 24.05	29.21 ± 0.60	1.86 ± 0.03

## Data Availability

The original contributions presented in the study are included in the article; further inquiries can be directed to the corresponding author.
